# The Neural Blueprint of Novelty: A Meta‐Analytic Dissection of Active and Passive Novelty Processing Networks

**DOI:** 10.1002/brb3.71221

**Published:** 2026-02-09

**Authors:** Ern Wong, Gianluca Sesso, Irene Sánchez Rodríguez, Jordi Manuello, Pietro Pietrini

**Affiliations:** ^1^ IMT School for Advanced Studies Lucca Molecular Mind Lab Lucca Italy; ^2^ IRCCS Stella Maris Foundation Scientific Institute of Child Neurology and Psychiatry Pisa Italy; ^3^ GCS‐fMRI Research Group, Koelliker Hospital, and Department of Psychology University of Turin Turin Italy; ^4^ Functional Neuroimaging and Complex Neural Systems Laboratory, Department of Psychology University of Turin Turin Italy

**Keywords:** Activation Likelihood Estimation (ALE), fMRI, medial temporal lobe, meta‐analysis, meta‐analytic connectivity modeling (MACM), novelty detection

## Abstract

**Background:**

Detecting novel environmental events is a fundamental survival mechanism, enabling organisms to identify and respond to salient changes. This function can operate in at least two broad modes, differing in task demands: active and passive novelty processing. Active processing involves explicitly recognizing novel or deviant stimuli and engaging goal‐directed, top‐down attentional control and memory‐related systems. In contrast, passive processing is driven primarily by bottom‐up attentional reorienting and does not necessarily require an explicit response or conscious evaluation. The present study asked whether these two modes recruit a shared neural architecture across task demands.

**Methods:**

We conducted a coordinate‐based meta‐analysis using Activation Likelihood Estimation (ALE) across fMRI studies of active and passive novelty processing. Conjunction and subtraction analyses were performed on the resulting ALE maps to identify common and distinct neural substrates associated with each mode of novelty processing.

**Results:**

The conjunction analysis revealed a core novelty‐responsive network encompassing the bilateral medial temporal lobes (MTLs), inferior frontal gyrus (IFG), and medial frontal regions. Subtraction analyses further identified task‐dependent specializations: studies of active novelty processing showed greater spatial convergence in the left precentral gyrus, left IFG, right MTL, and medial frontal areas, whereas studies of passive processing showed greater convergence in the left superior temporal gyrus, bilateral MTL, and right IFG.

**Conclusion:**

These findings suggest that active and passive conditions share a common novelty‐responsive network but differentially weight its components, reflecting distinct cognitive and attentional demands imposed by the explicit versus incidental processing of novel events.

## Graphical abstract

An ALE meta‐analysis of fMRI studies reveals a shared novelty‐responsive core (bilateral MTL, IFG, medial frontal cortex). Explicit, task‐relevant processing (active) emphasizes left prefrontal/motor and right MTL, whereas incidental, task‐irrelevant processing (passive) emphasizes salience regions, indicating task‐dependent spatial convergence within a common network.

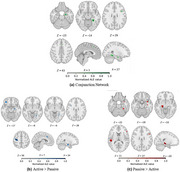

## 1 Introduction

Novelty detection— the ability to recognize and process new or unexpected stimuli— is a fundamental component of adaptive behavior. It enables organisms to prioritize salient information that is critical for survival, learning, and decision‐making by engaging neural systems responsible for rapid sensory evaluation, memory encoding, and assignment of motivational salience (Tiitinen et al. 1994; Ranganath and Rainer 2003). Aberrant novelty detection and approach have been linked to maladaptive behaviors in psychiatric conditions such as ADHD and schizophrenia (Lee et al. 2008; Kropotov et al. 2019; Collier et al. 2014). Despite extensive research, inconsistencies remain in the neural architecture reported for novelty detection, likely due to differences in experimental paradigms, imaging methods, and analytic approaches. This variability motivates the need for a quantitative synthesis to identify neural patterns that robustly support novelty processing across tasks and contexts.

The terms *novelty* and *surprise* are often used interchangeably, but they refer to related yet distinct computational constructs. Novelty is typically defined as the degree to which a stimulus or event has not been encountered before, or lacks an existing representation in memory. Surprise, by contrast, reflects a violation of expectations about what is likely to occur in a given context and is often formalized as a prediction error in probabilistic models (Barto et al. 2013; Schomaker et al. 2021; Modirshanechi et al. 2023; Monosov 2024). In many experimental paradigms, novel events are also surprising because they occur with low probability relative to an established context. Standard novelty tasks, therefore, rarely isolate novelty from surprise; instead, they engage a combination of novelty‐ and surprise‐related computations.

A further complication is that novelty itself is not a unitary concept. Theoretical work distinguishes at least two important forms: *stimulus* (or absolute) novelty and *contextual* (or relational) novelty (Schomaker et al. 2021; Schomaker and Meeter 2015). Stimulus novelty refers to items for which no prior memory trace exists—for example, trial‐unique images or pseudo‐objects that have not been seen before. Contextual novelty, in contrast, arises when a familiar stimulus violates expectations based on the current context or learned regularities, as in oddball or mismatch paradigms, where rare deviants appear within a stream of standard stimuli. In practice, most paradigms combine these dimensions to some extent; for instance, rare deviants in oddball tasks are often both relatively unfamiliar and highly unexpected. As a consequence, the associated neural responses likely reflect a mixture of novelty‐, surprise‐, and mismatch‐related processes. Throughout this work, we therefore use “novelty detection” as a label at the level of experimental design, while explicitly acknowledging that the underlying neural signals reflect both stimulus novelty and context‐dependent violations of expectation.

Within this conceptual framework, novelty detection is not a single, monolithic process but comprises at least two interrelated components based on task demands: an evaluative, top‐down component (“novel what?”) that involves recognizing and categorizing novel stimuli, and an orienting, bottom‐up component (“novel—now what?”) that directs attention toward unexpected events. This distinction maps onto two broad modes of novelty processing that we refer to here as *active* and *passive* novelty. We use *active novelty* to denote paradigms in which responses to novel or deviant events are explicitly task‐relevant and require a behavioral judgement (e.g., explicit novelty decisions, old/new recognition), and *passive novelty* to refer to paradigms in which novel or deviant stimuli are presented while attention is directed elsewhere or no explicit response to the deviant event is required, as in many oddball tasks (Brinkman and Stauder 2008; Justen and Herbert 2018). Importantly, these “passive” paradigms are more accurately characterized as involving task‐irrelevant, surprising deviant events rather than isolated stimulus novelty. Accordingly, our current approach is best understood as examining how task demands modulate the processing of deviant, novel/surprising events.

Early neuroimaging studies on novelty processing consistently implicate the medial temporal lobe (MTL), particularly the hippocampus and parahippocampal gyrus (PHG), across both active and passive paradigms. These observations motivate the “novelty‐encoding hypothesis” (Tulving et al. 1994), which posits that the brain's assessment of novelty is crucial in determining the effectiveness of long‐term memory encoding. Supporting this perspective, hippocampal responses to novelty appear to be automatic and can arise independently of explicit encoding intentions (Jessen et al. 2002). Furthermore, research has demonstrated that novelty processing generates a more robust hippocampal signal than deliberate encoding instructions (Menon et al. 2000). Taken together, these findings suggest that the hippocampus and adjacent MTL structures function as comparators or “mismatch detectors,” registering when incoming information is unfamiliar or deviates from stored representations, thereby signaling the need to update memory.

Electrophysiological studies converge on a similar picture. Event‐related potential (ERP) research has identified the P300 wave, particularly its P3a subcomponent, as a neural correlate of novelty processing. The P3a is a characteristic orienting response that peaks roughly 300 ms after novel stimulus onset and shows a frontoparietal scalp distribution (Courchesne et al. 1975; Friedman et al. 2001). Its amplitude declines with repeated exposure to a formerly novel stimulus, consistent with habituation (Courchesne et al. 1975). Critically, patients with hippocampal lesions exhibit impaired P3a habituation (Knight and Scabini 1998), suggesting that the hippocampus plays a crucial role in registering that a once‐novel stimulus has become familiar, possibly through comparisons with existing memory traces. Similar abnormalities in novelty P3 responses have been observed in patients with damage to the prefrontal cortex (PFC) (Yamaguchi and Knight 1991), indicating a broader network that supports novelty detection.

In active novelty detection tasks, such as explicit memory recognition tests, the brain engages a network of memory and control regions to discriminate novel from familiar stimuli. A meta‐analysis of 48 fMRI studies comparing “new” to “old” recognition events (Kim 2013) identified the MTL as the most consistently involved region. In addition, frontoparietal cognitive control regions, including the dorsolateral and dorsomedial PFC and intraparietal sulcus (IPS), are reliably involved during recognition, reflecting the cognitive effort involved in memory search and decision‐making. However, these regions tend to be engaged for both novel and familiar stimuli, suggesting a general role in retrieval effort rather than novelty‐specific processing. Thus, active novelty detection appears to rely on the hippocampal MTL system for encoding and discriminating new items, together with frontoparietal networks that support evaluative processes and decision‐making.

By contrast, passive novelty detection tasks such as oddball paradigms do not require participants to explicitly identify novel stimuli; yet, novel stimuli still elicit robust neural responses. A large meta‐analysis of 75 oddball fMRI studies (Kim 2014) demonstrated that novel or unexpected stimuli consistently recruit the ventral frontoparietal attention network, commonly associated with the salience network. Key regions include the temporoparietal junction (TPJ), bilateral IFG/insula, and dorsal anterior cingulate cortex (dACC). These regions are activated by deviant stimuli across auditory and visual modalities, suggesting a supramodal alerting system for unexpected events. Importantly, activation was more consistent when the oddball was task‐relevant, consistent with an interaction between bottom‐up saliency detection and top‐down attentional goals. The IFG, which also participates in dorsal attention and control networks, exhibits reliable convergence across oddball tasks, consistent with its role in task‐set updating and attention reorientation.

Beyond frontoparietal networks, both active and passive novelty tasks engage sensory and subcortical structures. Novel stimuli often enhance activity in modality‐specific sensory cortices, reflecting the early processing of deviance. For example, auditory oddball stimuli elicit increased activation in the auditory cortex, consistent with pre‐attentive mechanisms that amplify deviant signals (Downar et al. 2002). Subcortical regions, including the thalamus and basal ganglia, also make important contributions. The thalamus is thought to mediate sensory gating within corticothalamic loops, regulating the flow of novel information (Han et al. 2023). The striatum, particularly the ventral striatum, contributes to the assignment of motivational significance and to reinforcement learning, and has been implicated in determining whether novel stimuli are behaviorally relevant (Kafkas and Montaldi 2018; Wittmann et al. 2008; Guitart‐Masip et al. 2010). Early fMRI studies (Kiehl et al. 2001a, 2001b; Kiehl et al. 2005) in healthy individuals demonstrated that novel oddballs activate a widespread novelty network, including the bilateral TPJ, insula, superior temporal cortex, sensorimotor cortex, lateral PFC, dACC, thalamus, and striatum, underscoring the integrative nature of passive novelty processing.

Taken together, existing work suggests that there are partially overlapping yet distinct neural circuits for active and passive forms of novelty processing. Medial temporal regions appear to be engaged across a wide range of paradigms, consistent with a role in comparing incoming input to stored representations. At the same time, tasks that require explicit novelty judgements or memory decisions additionally recruit executive and motor regions associated with controlled retrieval and response selection. In contrast, paradigms in which novel or deviant events are task‐irrelevant more strongly engage attention networks that support stimulus‐driven orienting. What is currently lacking, however, is a comprehensive quantitative assessment of the common and distinct neural substrates of these two modes of novelty processing within a single framework.

The present study addresses this gap using coordinate‐based meta‐analysis and meta‐analytic connectivity modeling (MACM) to characterize how task demands shape the neural architecture of novelty processing across a broad range of paradigms. By directly contrasting active and passive conditions, we aim to identify (1) a core set of regions that is consistently engaged across novelty tasks and (2) task‐dependent specializations that distinguish deliberate, goal‐directed evaluation from more automatic, stimulus‐driven processing. In doing so, we bring together previously separate literatures on explicit, task‐relevant novelty evaluation (e.g., recognition and target‐detection tasks) and more incidental or task‐irrelevant processing of novel and deviant events (e.g., oddball, adaptation, and passive‐viewing paradigms), and provide a more integrative account of the neural systems that support novelty processing in the human brain.

## 2 Methods

### 2.1 Literature Search, Screening, and Extraction

This systematic review was conducted in accordance with the Preferred Reporting Items for Systematic Reviews and Meta‐Analyses (PRISMA) guidelines, with the protocol pre‐registered in PROSPERO (Registration No. CRD42024620192) to ensure methodological transparency. A comprehensive literature search was performed in PubMed and Scopus from inception to November 28, 2024, using the search query: (Novelty OR “novel stimuli” OR “novelty detection” OR “novelty processing” OR “novel decision” OR “novelty perception”) AND (“fMRI” OR “functional magnetic resonance imaging”). No restrictions were placed on the year of publication or geographical location, but only peer‐reviewed articles published in English were considered.

For qualitative synthesis, included studies met the following criteria (Manuello et al. 2022; Müller et al. 2018): (1) studies conducted on healthy adult participants with no history of neurological or psychiatric disorders; (2) empirical studies investigating novelty detection, explicitly contrasting a novelty condition against a baseline or control condition; (3) studies utilizing functional magnetic resonance imaging (fMRI) to examine brain activity; (4) studies reporting whole‐brain voxel‐wise activation results rather than region‐of‐interest (ROI)‐restricted analyses; and (5) studies providing activation coordinates in a stereotactic coordinate system, either Talairach or Montreal Neurological Institute (MNI) three‐dimensional (3D) space. Coordinates reported in Talairach space were converted to MNI space using the Lancaster transform (icbm2tal) incorporated in GingerALE (Laird et al. 2010; Lancaster et al. 2007).

Conversely, studies were excluded if they met any of the following criteria: (1) review articles, meta‐analyses, conference abstracts, or opinion papers that did not present original empirical data; (2) single‐case studies or case series; (3) longitudinal or interventional studies, unless they reported a baseline task‐based contrast relevant to novelty detection; (4) studies employing functional connectivity analyses instead of direct activation‐based contrasts; (5) studies based exclusively on ROI analyses without whole‐brain voxel‐wise activation results; and (6) studies involving clinical, neurological, or psychiatric populations.

Three authors (E.W., G.S., and I.S.R.) independently screened by title and abstract, selected articles for full‐text review, and performed full‐text reviews. Any disagreements that arose between the reviewers were resolved through discussion. The following information was extracted: authors, publication year, type of study design, number of participants by sex, age, and stereotactic coordinates. If multiple related contrasts were reported, we included all eligible contrasts but treated them as a single experiment, using only one set of coordinates per sample in the meta‐analysis (Turkeltaub et al. 2012). If further information was needed, authors were contacted at least twice via email, after which the data were considered irretrievable.

### 2.2 Active and Passive Classification

In the present meta‐analysis, we categorized experimental paradigms into active and passive novelty processing based on the level of participant engagement and explicit task demands. Active novelty processing was defined as paradigms in which participants were explicitly instructed to encode, categorize, or make decisions about novel stimuli, often with the expectation of a subsequent memory test or a direct novelty‐related judgment (e.g., old‐new recognition tasks, novelty‐based decision‐making, and categorization tasks). These paradigms require higher‐order cognitive engagement, including executive control and memory retrieval. On the contrary, passive novelty processing included paradigms where novelty was presented incidentally, and participants were not required to make explicit novelty‐related judgments or engage in directed encoding strategies (e.g., oddball paradigms, passive viewing, and incidental encoding tasks). In these studies, novelty detection is thought to rely more on bottom‐up, automatic salience‐driven neural mechanisms rather than goal‐directed processing. This classification enables a more precise comparison of the neural mechanisms underlying novelty detection across different cognitive and perceptual contexts, allowing us to distinguish between automatic novelty‐driven responses and effortful, top‐down novelty processing in the human brain.

### 2.3 Activation Likelihood Estimation

We used the Activation Likelihood Estimation (ALE) algorithm, as implemented in GingerALE v3.0.2 (Eickhoff et al. 2012, 2009; Turkeltaub et al. 2012), to perform the coordinate‐based meta‐analysis. Statistically significant foci from contrasts of interest were extracted and recorded for each study. This method tests for spatial convergence of coordinates across studies by modeling a 3D Gaussian probability distribution over reported foci to estimate the activation likelihood. The widths of the probability distributions are based on empirical estimates of spatial uncertainty in neuroimaging results due to sample size and between‐template variability, where a larger sample size assumes more reliable approximates of actual activation and is therefore associated with narrower distributions. These spatial probabilities were combined to obtain Modeled Activation (MA) maps of each experiment by taking the maximum probability associated with each focus. The final ALE image was obtained by combining the MA maps across studies. ALE scores were assessed against the null distribution of random spatial associations expected by chance to further distinguish signal from noise. ALE statistics were computed at each voxel for each dataset (general, active, and passive novelty detection). We applied cluster‐level family‐wise error correction to the resulting ALE maps (Eickhoff et al. 2016) to correct for multiple comparisons. A voxel‐level threshold of *p* < 0.001 (uncorrected) was used to define clusters, and a cluster‐level threshold of *p* < 0.05 was applied (Eickhoff et al. 2016; Müller et al. 2018). Statistical significance was determined using 1000 permutation tests to generate null distributions for cluster sizes (Müller et al. 2018).

The present meta‐analysis also aimed to identify brain regions that are robustly involved in novelty detection across studies and to differentiate between convergent and divergent neural substrates of active and passive novelty processing in healthy individuals. To achieve this, we conducted a series of contrast and conjunction analyses using ALE to compare the meta‐analytic maps for active and passive novelty detection. Our methodological approach followed these steps: (1) dataset analyses were conducted separately for active and passive novelty detection; (2) the datasets for active and passive novelty detection were pooled together using the “Merge & Save Foci” function in GingerALE; (3) a dataset analysis was conducted on the pooled foci file using identical statistical parameters as in the preceding analyses; and (4) the separate dataset analyses yielded three ALE maps representing general novelty detection, active novelty detection, and passive novelty detection, respectively.

The resulting ALE images were subjected to conjunction and contrast analyses within the GingerALE toolbox (Eickhoff et al. 2011) to determine common and distinct brain regions. The conjunction analysis identified shared neural substrates between active and passive novelty detection, representing domain‐general novelty processing. Conversely, contrast analyses were conducted to determine differentially activated regions specific to active versus passive novelty detection, highlighting domain‐specific neural mechanisms underlying each novelty detection process. By previous studies employing ALE‐based contrast and conjunction analyses (Gan et al. 2022), the ALE maps were thresholded at *p* < 0.05 (uncorrected) with a minimum cluster size of 100 mm^3^ and 10,000 permutations.

### 2.4 Functional Decoding

We employed a meta‐analytic decoding approach using the Brain Annotation Toolbox (BAT) (Liu et al. 2019) to facilitate a comprehensive functional characterization of the identified brain regions. BAT is a meta‐analytic tool based on activation maps in MNI space for 217 functional terms derived from the Neurosynth database (Yarkoni et al. 2011). The toolbox provides a probabilistic framework to infer functional relevance by assessing whether voxels within a given cluster or region are more likely to be coactivated with specific functional terms than randomly selected voxels.

This approach enables functional characterization based on meta‐analytically associated terms, allowing for a data‐driven interpretation of the identified regions’ potential roles (Liu et al. 2019). In the present study, we used BAT to decode functional associations for core novelty detection, which was determined through the conjunction between active and passive networks. Statistical significance was determined using 10,000 permutations, with a *p <* 0.05 threshold to ensure robust inference.

### 2.5 Meta‐Analytic Connectivity Modeling

We conducted MACM using Sleuth v3.0.4 and GingerALE v3.0.2 to further investigate the broader task‐dependent functional connectivity underlying novelty detection. While our primary ALE meta‐analyses focused explicitly on studies of novelty detection, MACM enables the characterization of more general coactivation profiles by leveraging a much wider range of task‐based neuroimaging studies. This allows us to examine whether the regions jointly involved in novelty processing form a functionally coherent network across diverse cognitive domains.

Seed regions for the MACM analysis were derived from the ALE conjunction analysis of active and passive novelty detection. These significant clusters reflect regions commonly involved in both forms of novelty processing. To avoid redundancy due to spatial overlap—particularly in densely clustered areas such as the right hippocampus—we selected only the peak coordinate with the highest ALE value within each cluster. This procedure yielded seven nonoverlapping seed regions for subsequent MACM analysis. MACM identifies regions that consistently coactivate with a given ROI across multiple task‐based studies, thereby revealing task‐dependent functional connectivity (Eickhoff et al. 2011; Laird et al. 2013). Unlike resting‐state connectivity, which captures intrinsic, spontaneous neural fluctuations, MACM reflects coactivation under controlled task conditions, providing insight into the functional roles of regions across various cognitive contexts. Importantly, standard ALE analyses—though informative—do not guarantee direct functional connectivity between regions. For instance, two areas (e.g., A and B) may appear in the same ALE map simply because they both coactivate with a third region (C), without any direct coactivation between A and B. MACM addresses this limitation by including only studies that report activation within the seed ROI itself, ensuring that identified coactivations reflect direct statistical associations with the seed region.

For each seed ROI (defined as a 6 mm‐radius sphere around the peak coordinate), we queried the BrainMap database using the following search criteria: (1) *Activations*: activations only, (2) *Context*: normal mapping, (3) *Subject Diagnosis*: healthy controls. Studies meeting these criteria were extracted and analyzed in GingerALE, applying an ALE threshold of *p* < 0.01 (uncorrected) with a minimum cluster volume of 250 mm^3^. The resulting MACM ALE maps were statistically thresholded and visualized to examine significant coactivation patterns. This procedure provides a standardized way to characterize the task‐based coactivation profile of the ALE peak across the BrainMap database (Eickhoff et al. 2011; Laird et al. 2013). Importantly, for subcortical structures such as the hippocampus, the spherical ROI should be interpreted as a local seed region centered on the peak rather than as an approximation of the anatomical structure as a whole.

To construct a functional connectivity network model from the MACM results, we operationalized nodes as the seven peak ALE coordinates derived from the conjunction analysis. Although ALE and MACM operate at the voxel level, each seed ROI (centered on its peak voxel) was treated as a distinct node for network modeling purposes. For each pair of ROIs, we assessed whether the MACM for one ROI showed significant coactivation within the spherical ROI of the other. This analysis was repeated in both directions to evaluate the strength and directionality of coactivation (Kotkowski et al. 2018; Li et al. 2025).

Coactivation strength between nodes was quantified using Bonferroni‐corrected *p*‐values to control for multiple comparisons. The correction factor was applied across all pairwise comparisons, yielding a significance threshold of *p* = 0.00714. Based on these corrected values, we constructed an adjacency matrix representing the connectivity structure. Edges were classified as unidirectional if significant coactivation was observed in only one direction (e.g., ROI A *→* ROI B) and bidirectional if both directions reached significance. This meta‐analytic network model offers a task‐dependent representation of functional interactions among regions implicated in novelty processing. However, we emphasize that this approach does not permit inferences about causality or directional influence between brain regions.

## 3 Results

**FIGURE 1:**
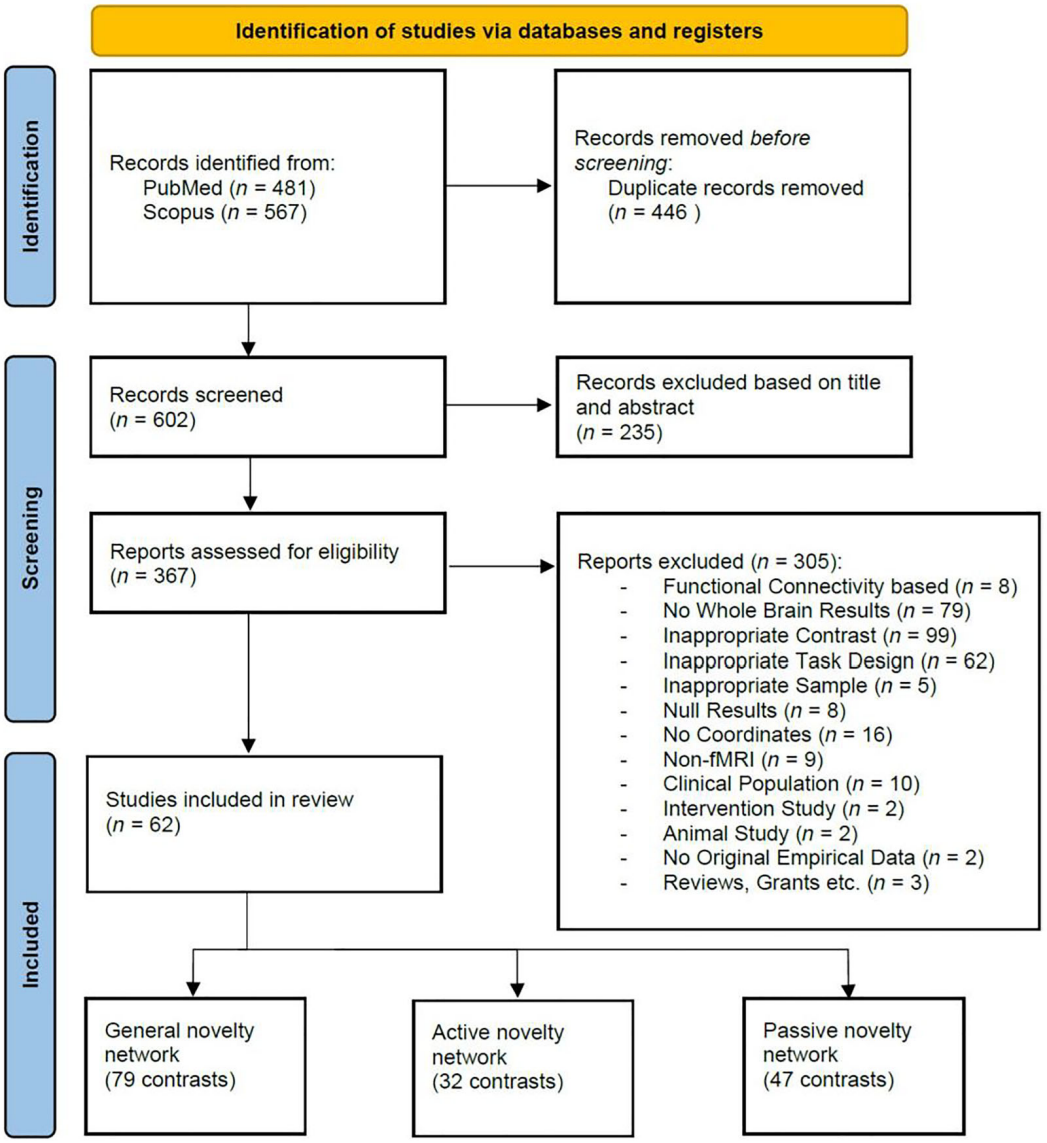
PRISMA flowchart illustrating article screening, exclusion, and inclusion processes according to guidelines.

A total of 602 papers were identified after removing duplicates, and 235 papers were excluded during title and abstract screening, leaving 367 papers for full‐text screening. A final 62 papers were included in the current meta‐analysis. Out of these 62 papers and after pooling related contrasts, 66 experiments were identified, with 27 experiments classified under active and 39 experiments under passive novelty detection (Figure 1, Table S1).

### 3.1 General Novelty Detection

**FIGURE 2:**
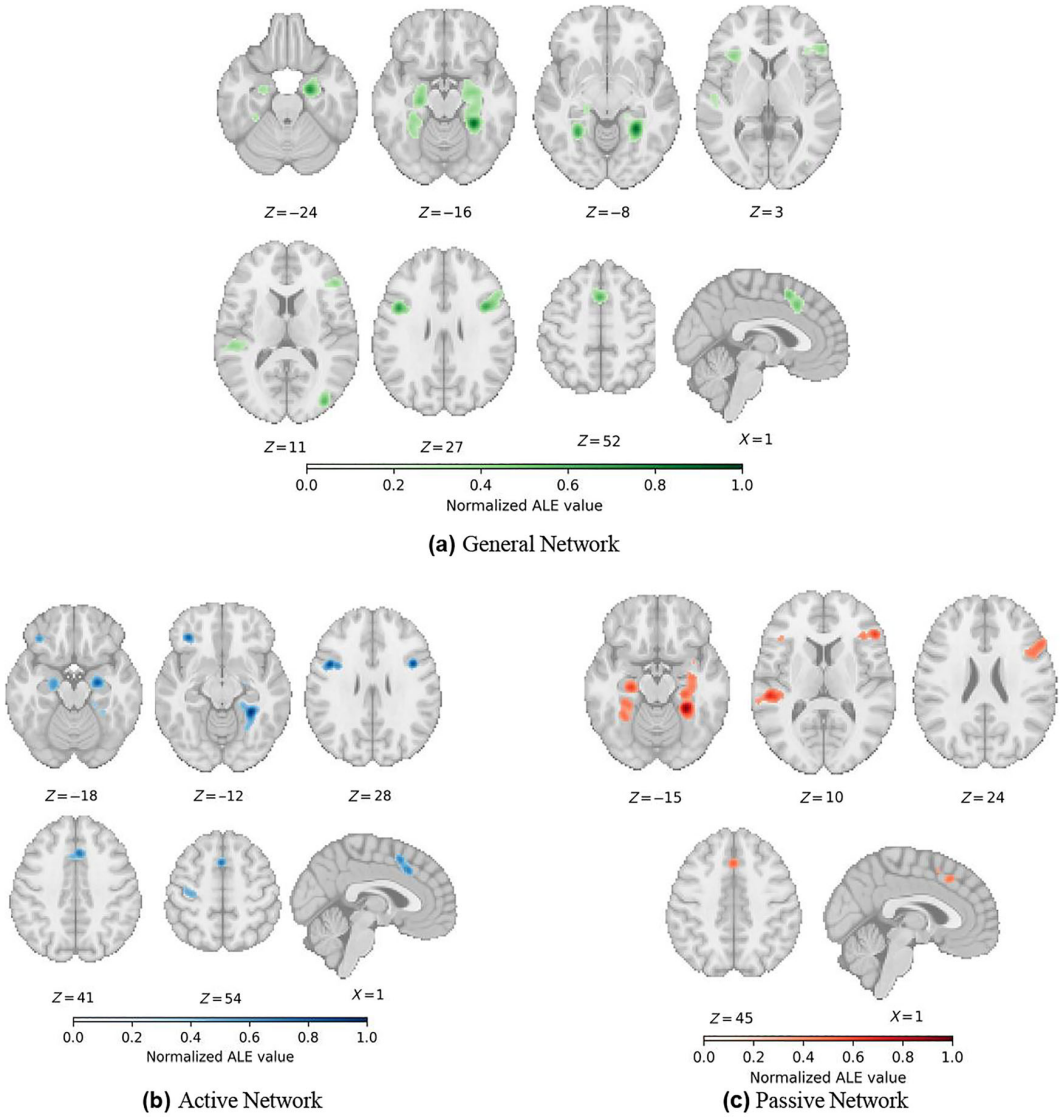
Overview of significant clusters from dataset analyses in healthy subjects. (a) General network of all included studies, (b) network of studies classified under active novelty detection, and (c) network of studies classified under passive novelty detection. Coordinates are in MNI space.

The ALE meta‐analysis of 66 experiments (1041 foci, 1771 subjects) identified eight significant clusters associated with novelty detection. A cluster in the right MTL spanned the hippocampus, PHG, and thalamus, while a left MTL cluster extended into the fusiform gyrus, PHG, and amygdala. Additional clusters were observed in the right lateral frontal cortex, including the precentral gyrus, IFG, middle frontal gyrus (MFG), and insula. A bilateral cluster was identified in the medial frontal wall, encompassing the superior and medial frontal gyrus that overlapped the supplementary motor area (SMA). Further clusters were found in the right middle occipital gyrus (MOG), left superior temporal gyrus (STG), left IFG, and left insula (Table 1, Figure 2).

**TABLE 1:** ALE results for general novelty detection.

Volume (mm^3^)	Side	Brain region	BA	MNI coordinates			ALE (10‐2)	*Z*
				*x*	*y*	*z*		
12,856	R	Parahippocampal Gyrus	36	28	−44	−12	8.07	9.37
	R	Parahippocampal Gyrus	34	22	−10	−24	6.07	7.65
	R	Hippocampus		32	−22	−18	3.27	4.83
	R	Parahippocampal Gyrus	35	28	−28	−16	3.24	4.79
	R	Thalamus		20	−30	−2	2.47	3.86
	R	Superior Temporal Gyrus	38	36	4	−20	2.29	3.63
8304	L	Fusiform Gyrus	37	−28	−52	−10	5.39	7.01
	L	Parahippocampal Gyrus	28	−24	−20	−16	4.23	5.86
	L	Parahippocampal Gyrus	36	−30	−36	−20	4.07	5.7
	L	Amygdala		−22	−12	−18	3.74	5.35
	L	Amygdala		−24	−8	−28	3.08	4.61
	L	Parahippocampal Gyrus	28	−20	−28	−8	2.5	3.91
6488	R	Precentral Gyrus	6	42	8	28	5.29	6.92
	R	Inferior Frontal Gyrus	13	50	30	8	3.74	5.35
	R	Middle Frontal Gyrus	9	54	20	24	3.57	5.16
	R	Middle Frontal Gyrus	46	48	32	16	3.45	5.03
	R	Insula	13	38	28	8	2.77	4.24
4360	L	Superior Frontal Gyrus	6	0	12	52	4.75	6.4
	R	Medial Frontal Gyrus	6	2	20	42	4.11	5.74
2368	R	Middle Occipital Gyrus	19	36	−84	12	4.47	6.11
2336	L	Superior Temporal Gyrus	41	−52	−30	10	3.42	5
	L	Superior Temporal Gyrus	22	−54	−16	4	2.44	3.83
2200	L	Inferior Frontal Gyrus	9	−44	6	28	5.66	7.26
1232	L	Insula	13	−34	24	2	3.64	5.24

### 3.2 Active Novelty Detection

The ALE analysis of 27 experiments (330 foci, 992 subjects) for active novelty detection revealed eight significant activation clusters. Three clusters were identified in the bilateral MTL, with two clusters in the right PHG and one in the left PHG extending into the amygdala. A bilateral cluster was observed in the medial frontal wall, spanning the superior frontal gyrus (SFG), including the SMA and anterior cingulate gyrus (CG). Additional significant clusters were found in bilateral IFG and precentral gyri (Table 2, Figure 2).

**TABLE 2:** ALE results for active novelty detection.

Volume (mm3)	Side	Brain region		MNI coordinates			ALE (10‐2)	Z
				*x*	*y*	*z*		
3512	R	Parahippocampal Gyrus	36	30	−42	−12	3.79	6.47
	R	Parahippocampal Gyrus	37	30	−46	−8	3.74	6.42
	R	Cerebellum		22	−36	−14	2.13	4.39
2816	R	Parahippocampal Gyrus	34	24	−12	−22	3.69	6.35
2664	L	Superior Frontal Gyrus	6	−2	12	52	3.11	5.67
	R	Cingulate Gyrus	32	4	20	40	2.85	5.34
1592	L	Inferior Frontal Gyrus	9	−44	6	28	3.36	5.97
	L	Precentral Gyrus	6	−38	6	28	2.53	4.93
1336	L	Parahippocampal Gyrus	28	−20	−14	−18	2.29	4.6
	L	Amygdala		−24	−8	−28	1.76	3.86
1128	L	Precentral Gyrus	4	−38	−18	56	2.25	4.55
	L	Precentral Gyrus	4	−32	−22	56	2.18	4.46
1008	L	Inferior Frontal Gyrus	47	−34	34	−14	3.53	6.17
832	R	Precentral Gyrus	6	42	8	28	3.42	6.04

### 3.3 Passive Novelty Detection

The ALE analysis of 39 experiments (711 foci, 779 subjects) for passive novelty detection identified six distinct activation clusters. Two large clusters were found in the bilateral MTL, with the right cluster spanning the hippocampus, PHG, and amygdala, while the left cluster extended across the fusiform and parahippocampal regions. An extensive cluster was identified in the right lateral frontal lobe, spanning the IFG, middle frontal gyrus, and insula. A cluster in the medial frontal wall corresponding to the SMA was also observed. Additionally, activation was found in the left STG and a smaller cluster in the left IFG (Table 3, Figure 2).

**TABLE 3:** ALE results for passive novelty detection.

Volume (mm^3^)	Side	Brain region	BA	MNI coordinates			ALE (10‐2)	*Z*
				*x*	*y*	*z*		
7712	R	Parahippocampal Gyrus	36	28	−44	−14	4.96	7.39
	R	Amygdala		22	−8	−24	2.76	4.84
	R	Parahippocampal Gyrus	35	26	−28	−16	2.58	4.61
	R	Amygdala		32	−10	−20	2.41	4.38
	R	Hippocampus		34	−22	−18	2.37	4.34
	R	Superior Temporal Gyrus	38	36	4	−20	2.28	4.2
	R	Amygdala		26	2	−22	2.13	3.99
5856	L	Fusiform Gyrus	37	−30	−52	−10	3.52	5.79
	L	Parahippocampal Gyrus	36	−30	−36	−20	3.16	5.35
	L	Parahippocampal Gyrus	28	−24	−22	−16	3.03	5.2
	L	Cerebellum		−34	−48	−20	2.56	4.59
	L	Parahippocampal Gyrus	28	−20	−28	−8	2.49	4.5
5208	R	Inferior Frontal Gyrus	13	50	30	8	3.39	5.63
	R	Inferior Frontal Gyrus	9	48	12	28	3.2	5.41
	R	Middle Frontal Gyrus	9	54	20	22	2.53	4.55
	R	Middle Frontal Gyrus	46	50	30	18	2.33	4.28
	R	Insula	13	38	28	10	2.13	3.99
2224	L	Superior Temporal Gyrus	41	−52	−30	10	3.34	5.58
	L	Superior Temporal Gyrus	22	−64	−36	10	1.81	3.52
1248	L	Medial Frontal Gyrus	6	0	22	46	2.57	4.6
	L	Superior Frontal Gyrus	6	−6	18	54	2.46	4.46
	L	Superior Frontal Gyrus	6	0	12	54	1.8	3.51
1024	L	Insula	13	−36	24	4	2.26	4.18
	L	Inferior Frontal Gyrus	45	−42	26	12	2.08	3.92

### 3.4 Common and Distinct Brain Regions for Active vs. Passive Novelty Detection

**FIGURE 3:**
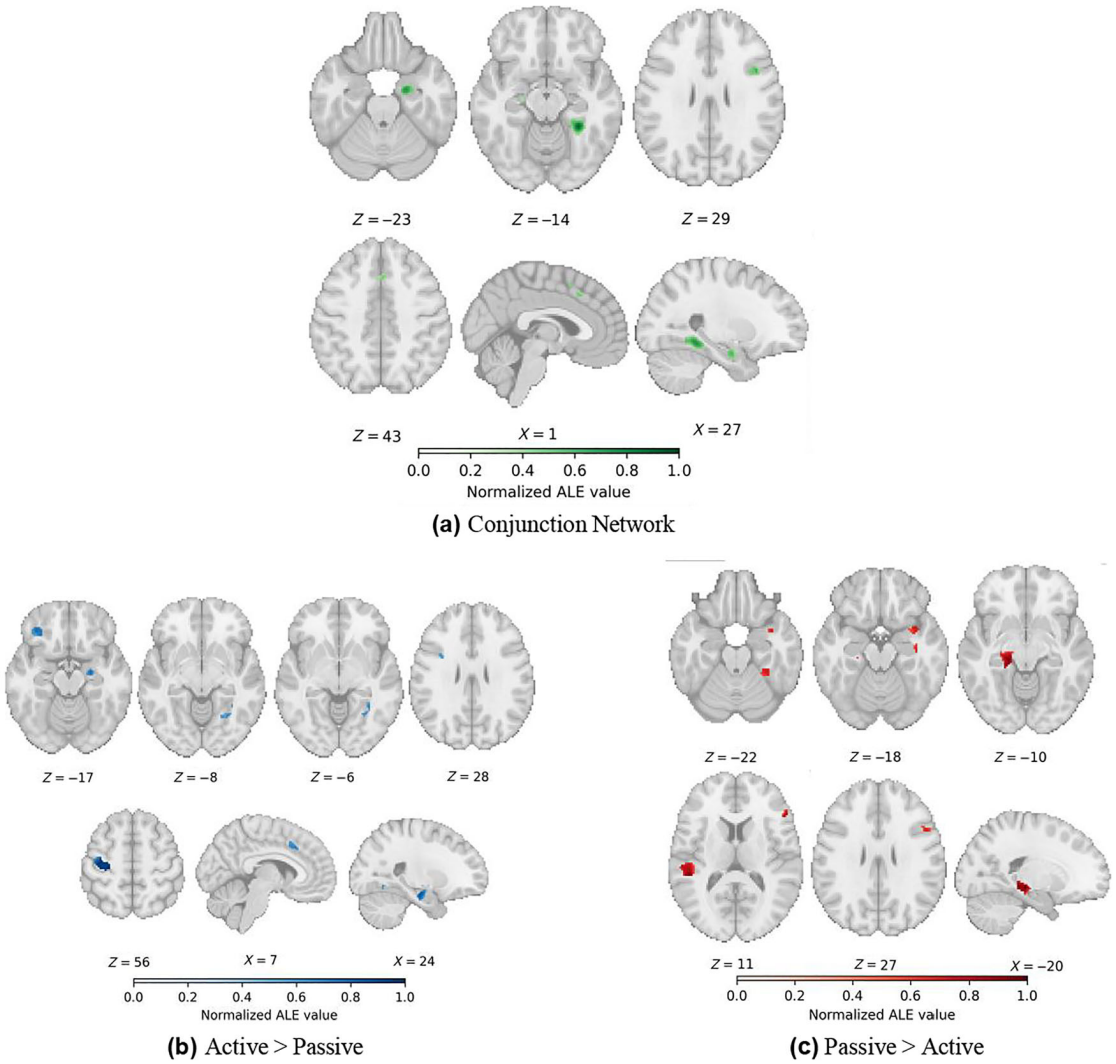
Overview of significant clusters from the conjunction and contrast analyses in healthy subjects. (a) Common activations for active and passive novelty detection, (b) greater activation convergence for active than passive novelty detection, and (c) greater activation convergence for passive than active novelty detection. Coordinates are in MNI space.

The meta‐analytical conjunction analysis was performed to identify the shared network between active and passive novelty detection, revealing eight significant activation clusters. Five clusters were located in the MTL, with the right MTL containing clusters in the PHG and amygdala, as well as two smaller clusters in the hippocampus. In the left MTL, one cluster was identified in the PHG. Two clusters were observed in the medial frontal wall, specifically in the left SFG (SMA and the left CG). Additionally, one cluster was identified in the right IFG (Table 4, Figure 3).

**TABLE 4:** Conjunction and contrast analysis for active and passive networks.

Volume (mm^3^)	Side	Brain region	BA	MNI coordinates			ALE (10‐2)	
				*x*	*y*	*Z*		
*Active Conj. Passive*								
1864	R	Parahippocampal Gyrus	36	30	−42	−12		3.79
1136	R	Amygdala		22	−8	−24		2.76
504	R	IFG	9	44	10	28		2.33
208	L	Parahippocampal Gyrus	28	−22	−18	−18		1.98
208	L	Superior Frontal Gyrus	6	0	12	54		1.8
176	L	Cingulate Gyrus	32	0	20	42		1.86
16	R	Hippocampus		30	−22	−20		1.47
8	R	Hippocampus		30	−18	−18		1.37
*Active > Passive*								
1040	L	Precentral Gyrus	4	−27.3	−24	55.3		3.06
	L	Precentral Gyrus	4	−35	−19	52		3.01
	L	Precentral Gyrus	4	−35.8	−16.7	57.8		2.99
	L	Precentral Gyrus	4	−28	−18	56		2.91
896	L	Inferior Frontal Gyrus	47	−36	35	−16		2.41
	L	Inferior Frontal Gyrus	47	−32	32	−18		2.26
720	R	Parahippocampal Gyrus	28	24	−14	−18		2.61
472	R	Medial Frontal Gyrus	32	10	18	42		2.34
248	R	Parahippocampal Gyrus	19	30	−58	−6		2.12
	R	Cerebellum		24	−62	−10		1.96
176	L	Precentral Gyrus	6	−34	8	30		2.12
128	R	Parahippocampal Gyrus	19	34	−50	−6		2.13
*Passive > Active*								
1416	L	Superior Temporal Gyrus	41	−48	−34	14		2.82
	L	Superior Temporal Gyrus	41	−50	−28	16		2.55
	L	Transverse Temporal Gyrus	41	−54	−26	14		2.52
	L	Superior Temporal Gyrus	22	−58	−30	8		2.37
1232	L	Parahippocampal Gyrus	28	−20	−26	−8		2.86
	L	Parahippocampal Gyrus	27	−21	−32	−8		2.78
1016	R	Inferior Frontal Gyrus	45	52	24	14		2.79
	R	Inferior Frontal Gyrus	45	54	28	12		2.59
	R	Inferior Frontal Gyrus	13	48	32	0		2.15
	R	Inferior Frontal Gyrus	9	54	18	18		1.91
352	R	Inferior Frontal Gyrus	9	52	12	24		2.18
	R	Middle Frontal Gyrus	9	50	14	32		2.04
304	R	Inferior Frontal Gyrus	13	34	6	−20		2.16
	R	Inferior Frontal Gyrus	13	38	6	−18		2.16
	R	Superior Temporal Gyrus	38	38	2	−20		2.09
184	R	Cerebellum		32	−40	−24		2.23
144	R	Hippocampus		38	−18	−16		2.16

The meta‐analytical contrast analysis was conducted to identify brain regions more specific to active and passive novelty detection. Active novelty detection showed greater recruitment in seven clusters, including three clusters in the right PHG, one in the left precentral gyrus, one in the left IFG, one in the medial frontal gyrus corresponding to the anterior CG, and one in a ventral portion of the precentral gyrus (Table 4, Figure 3).

For passive novelty detection, greater recruitment was observed in seven clusters. Two clusters were found in MTL regions, one in the left PHG and another in the right hippocampus. A large cluster was identified in the left STG, extending into the transverse temporal gyrus. Three clusters were observed in the right lateral frontal surface, with one in the IFG, another spanning the inferior and middle frontal gyri, and the final cluster in the insula extending into the STG. Additionally, one cluster was identified in the cerebellum (Table 4, Figure 3).

### 3.5 Meta‐Analytic Connectivity Modeling

MACM was conducted to examine the extent of connectivity between the seven distinct ROIs identified in the ALE conjunction analysis of the core novelty detection network. A separate MACM analysis was performed for each ROI, resulting in seven independent seed‐to‐voxel connectivity maps. Arrows were used to represent directionality, indicating that variance in one node was predictive of variance in another but not vice versa. However, these directed edges only summarize meta‐analytical connectivity patterns within the core novelty network and do not imply causal relationships between regions.

**FIGURE 4:**
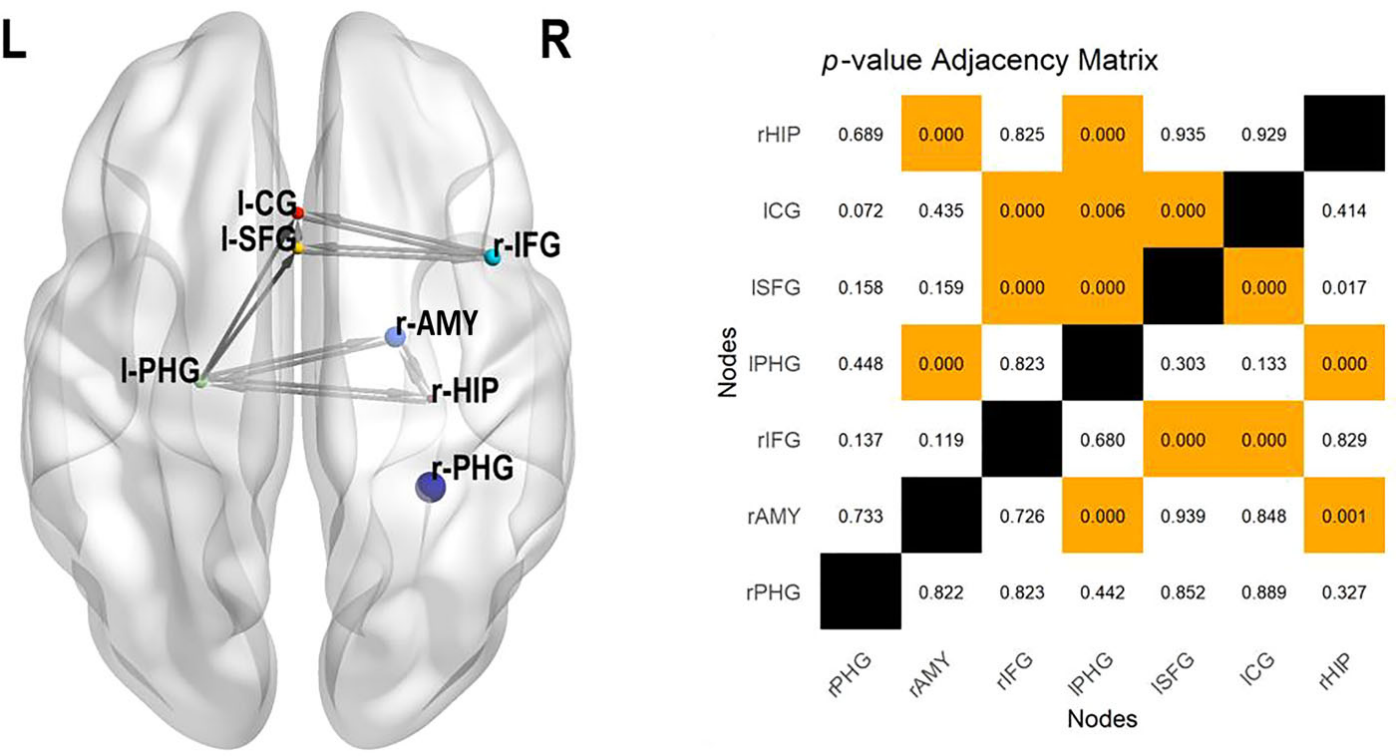
Left: Overview of meta‐analytic connectivity modeling (MACM) of the core novelty detection network, derived from the conjunction analysis of active and passive novelty detection. The network illustrates task‐based coactivation patterns among regions commonly implicated in both forms of novelty processing across cognitive domains. Node size is proportional to ALE values, with larger nodes indicating greater spatial convergence. Right: *p*‐value adjacency matrix representing connectivity structure derived from individual ROI MACM maps. Orange squares represent statistically significant edges after correction for multiple comparisons. l‐CG = left cingulate gyrus; l‐PHG = left parahippocampal gyrus; l‐SFG = left superior frontal gyrus; r‐AMY = right amygdala; r‐HIP = right hippocampus; r‐IFG = right inferior frontal gyrus; r‐PHG = right parahippocampal gyrus.

The analysis revealed bidirectional coactivations between the right amygdala, right hippocampus, and left PHG. Additionally, bidirectional coactivations were observed between the right IFG and both the left CG and left SFG. Directed coactivity was identified from the left PHG to the left CG and left SFG and from the left SFG to the left CG. No significant coactivation activity was observed for the right PHG (Figure 4).

### 3.6 Functional Decoding of Core Novelty Detection Network

**FIGURE 5:**
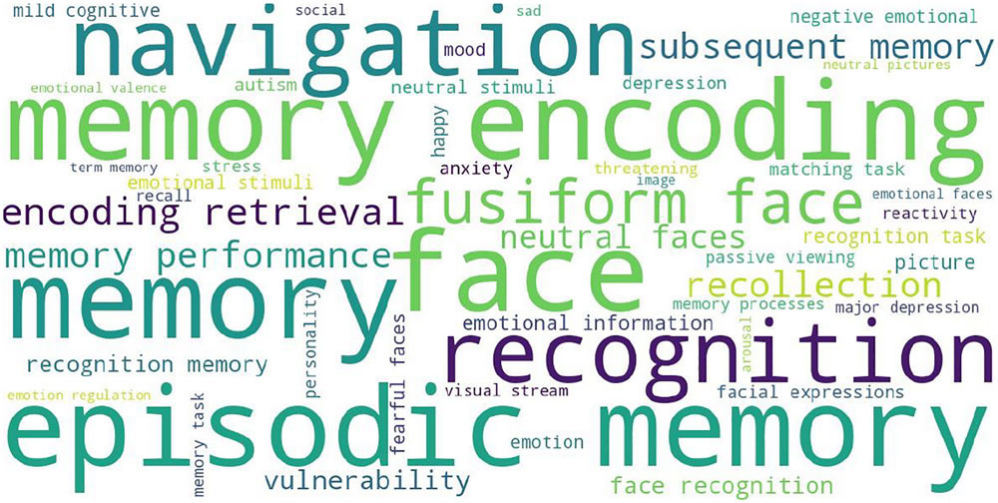
Functional decoding of the core novelty detection network, derived from the conjunction analysis of active and passive ALE meta‐analytical maps. The word cloud displays the top 50 functional terms associated with the network based on meta‐analytic coactivation patterns from Neurosynth. Font size is proportional to the statistical strength of association, with larger font sizes indicating smaller permutation‐based *p*‐values.

We characterized regions robustly engaged during core novelty detection, as defined by the conjunction analysis of active and passive novelty networks. Specifically, a quantitative functional annotation analysis was conducted to test the association between functional terms from the Neurosynth database and the identified brain regions. The results indicated that these regions were primarily associated with memory encoding, retrieval, and recognition processes (Figure 5).

## 4 Discussion

### 4.1 General, Active, and Passive Networks

This meta‐analysis asked how task demands shape the neural architecture of novelty processing. We focused on two broad classes of paradigms: *active* conditions, in which novel or deviant events were explicitly task‐relevant and required a behavioral judgement (for example, explicit novelty decisions, old/new recognition, or target detection), and *passive* conditions, in which novel or deviant stimuli were presented while attention was directed elsewhere or no explicit response was required, as in many oddball or passive‐viewing tasks. By comparing these two sets of studies, we aimed to identify both a core circuitry consistently engaged across novelty paradigms and task‐dependent specializations that distinguish deliberate, goal‐directed evaluation from more automatic, stimulus‐driven processing.

Across all studies, the ALE analysis revealed a distributed network associated with novelty processing, encompassing sensory cortices, MTL structures, and frontal salience and control regions. This pattern converges with previous work and reinforces the view that novelty processing does not rely on a single locus but instead emerges from coordinated interactions among systems supporting sensory analysis, comparison with stored regularities, attentional reorienting, and preparation of behavioral responses. Within this network, convergence in occipital and superior temporal regions likely reflects enhanced processing of deviant or unusual input in modality‐specific sensory areas (Downar et al. 2000). Thalamic convergence is consistent with its proposed role as a relay and gating hub within corticothalamic loops, routing deviant signals toward higher‐order integrative regions (Han et al. 2023). Convergence in MTL regions—including PHG, hippocampus, and amygdala—and in lateral and medial frontal regions points to a broader circuit that evaluates the significance of deviant events and supports adaptive behavioral adjustment (Barbeau et al. 2017; Lisman and Redish 2009; Mehta et al. 1997; Skaggs et al. 1996; Stachenfeld et al. 2017; Pezzulo et al. 2017; Corbetta and Shulman 2002; Yeo et al. 2011; Downar et al. 2002; Naeije et al. 2016).

Examining the active subset, our meta‐analysis aligns with prior findings, particularly a meta‐analysis investigating “new‐vs.‐old” effects, which demonstrated greater convergence in the anterior MTL, including hippocampus and amygdala (Kim 2013, 2021). Our results converge with this work, highlighting extensive bilateral convergence in PHG, hippocampus, and amygdala, regions crucial for novelty‐related memory encoding and contextual association formation. We also observed convergence in medial prefrontal regions, consistent with their role in linking novelty signals to action selection and performance monitoring. Beyond these shared patterns, active conditions showed stronger convergence in bilateral inferior frontal regions and in the left precentral gyrus. The left precentral gyrus, part of motor and premotor cortices, likely supports motor planning and response selection (Manzone and Welsh 2023; Kubanek et al. 2024), indicating that explicit novelty judgements place additional demands on preparatory motor engagement and overt response execution.

Although prior meta‐analyses have not systematically examined “passive novelty detection” as a separate construct, our passive subset showed substantial overlap with ALE studies on attentional reorienting and oddball detection (Kim 2014). The identified network corresponds broadly to the ventral attention/salience system, which underlies stimulus‐driven attentional shifts (Corbetta and Shulman 2002; Yeo et al. 2011). We observed robust convergence in bilateral insula and medial frontal regions, suggesting that passive paradigms are dominated by salience signals and automatic orienting. A notable deviation from previous oddball meta‐analyses is the reliable convergence we observed in hippocampus and parahippocampal cortex under passive conditions. Most earlier work did not report such effects, likely because many studies focused on target detection oddballs or used contrasts that collapsed across truly novel and merely deviant stimuli (Strange and Dolan 2001; Schomaker et al. 2021). In contrast, our screening distinguished novel and unexpected stimuli from targets and included a broader range of passive paradigms (e.g., adaptation designs and passive viewing), revealing consistent convergence in the MTL. This pattern suggests that memory‐based mismatch computations are engaged even when novelty is task‐irrelevant, and that passive novelty processing is not purely a function of bottom‐up attentional capture. Instead, automatic salience‐driven mechanisms and medial temporal mismatch processes appear to operate in parallel. We also observed convergence in auditory regions, likely reflecting the prevalence of auditory oddball paradigms in the passive corpus. By contrast, we did not find robust convergence in some regions traditionally associated with attentional reorienting, such as the TPJ and IPS. This absence may reflect our emphasis on novelty‐sensitive contrasts and our inclusion criteria, rather than indicating that parietal regions are uninvolved in orienting (Downar et al. 2000).

### 4.2 Common Network for Novelty Detection

The conjunction analysis identified a core novelty‐sensitive network common to both active and passive tasks, centering on the MTL, including the hippocampus and parahippocampal gyri. This aligns with earlier meta‐analytic findings on recognition memory (Kim 2013, 2021) and repetition suppression (Kim 2017), and extends them to a broader set of paradigms in which novel or deviant events are not always explicitly task‐relevant. Our findings reinforce the functional heterogeneity of the hippocampal–parahippocampal system in novelty‐related processing. Rather than supporting a dedicated, novelty‐specific module, the pattern is most consistent with predictive coding and mismatch accounts of MTL function. On these accounts, hippocampal circuitry generates expectations about upcoming input based on learned regularities and signals mismatches when actual input deviates from those expectations (Barbeau et al. 2017; Lisman and Redish 2009; Mehta et al. 1997; Skaggs et al. 1996; Stachenfeld et al. 2017; Pezzulo et al. 2017). Novel stimuli are a prominent source of such mismatches, but similar signals can be elicited by unexpected configurations of familiar elements. Our results, therefore, suggest that hippocampal and parahippocampal convergence in novelty tasks reflects these mismatch or prediction‐error computations, which are robustly engaged across both active and passive contexts.

Within the posterior MTL, the perirhinal cortex (BA36) also showed particularly strong convergence in the conjunction analysis. This dovetails with recent primate electrophysiological findings that neuronal populations in perirhinal cortex respond preferentially to novel objects, whereas neurons in hippocampus, basal forebrain, and striatum are more sensitive to unpredictability and violations of well‐learned sequences (Zhang et al. 2022; Monosov 2024). Taken together, these findings suggest a functional gradient in which perirhinal cortex contributes to assessing stimulus‐level novelty or familiarity, whereas hippocampal subregions play a larger role in encoding expectation violations and sequence learning errors.

Beyond the MTL, the IFG and dorsomedial prefrontal cortex (dmPFC)—encompassing SMA and dACC— emerged as key frontal components of the common network. The IFG has been identified as a critical node in networks supporting salience detection and attentional reorienting (Corbetta and Shulman 2002). It has been linked to both the salience and ventral attention networks, and is sensitive to both stimulus‐driven and goal‐directed demands (Bedini and Baldauf 2021; Brass et al. 2005), making it well‐suited to integrating deviance signals across a range of task contexts. The dmPFC is associated with conflict monitoring, error detection, and cognitive control regulation (Botvinick et al. 2001; MacDonald et al. 2000). It is consistently engaged in tasks requiring detection of novel or unexpected events, such as oddball paradigms (Braver et al. 2001), highlighting their role in assessing the behavioral significance of deviant stimuli and guiding adaptive responses. The dACC, in particular, integrates prediction errors and reinforcement learning signals via dopaminergic pathways (Holroyd and Coles 2002), which are critical for updating behavioral strategies based on novel or surprising information (Alexander and Brown 2011, 2019). The SMA, in turn, contributes to translating novelty‐related signals into action, adjusting motor sequences or inhibiting prepotent responses to prioritize adaptive behaviors (Nachev et al. 2008). Direct connectivity between the dACC and SMA, and their frequent coactivation during novelty processing (Picard and Strick 1996), supports a tight functional coupling between evaluation and motor preparation.

A notable observation is that, despite frequent conceptual links between novelty, reinforcement learning, and reward prediction‐error signaling, our ALE analyses did not reveal significant convergence in ventral striatum or ventromedial prefrontal cortex (vmPFC)—key nodes of canonical reward prediction‐error circuitry. We believe this absence reflects characteristics of the available literature and common reporting practices rather than evidence against the involvement of these regions. First, our inclusion criteria primarily captured perceptual novelty and oddball paradigms; relatively few studies explicitly linked novelty to reward outcomes or value‐based decisions, where ventral striatal and vmPFC responses are typically strongest (Wittmann et al. 2008; Guitart‐Masip et al. 2010; Kafkas and Montaldi 2018). Second, novelty‐ and surprise‐related effects in these regions appear highly task‐ and context‐dependent, which can produce spatially heterogeneous activations that do not converge robustly across paradigms. Third, many reinforcement‐learning studies rely on ROI or model‐based analyses and small‐volume corrections, so significant effects in ventral striatum/vmPFC are not always reported as whole‐brain peak coordinates suitable for ALE (Holroyd and Coles 2002). Accordingly, we interpret our findings as highlighting the most consistently reported cross‐paradigm engagement of medial temporal and frontoparietal regions, while remaining fully consistent with the broader literature that implicates the ventral striatum and vmPFC in value‐modulated aspects of novelty and surprise.

MACM of this network reveals strong coactivation between the MTL and key salience network nodes, including the right IFG, dACC, insula and motor cortices. This pattern suggests an underlying general orienting‐evaluation circuit, in which hippocampus and perirhinal cortex compute mismatches between expected and experienced events, frontal salience regions register their behavioral significance, and motor regions prepare appropriate responses. This integrative mechanism aligns with the concept of a central novelty‐processing hub that mediates interactions between memory and attentional control systems, ensuring effective adaptation to environmental change.

### 4.3 Differences Between Active and Passive Networks

The contrast analysis revealed different neural signatures for active and passive novelty detection, reflecting differences in their cognitive demands and attentional mechanisms. These align with theoretical models positing that active novelty detection relies on top‐down executive control and memory integration, whereas passive detection depends on bottom‐up salience signaling and sensory processing. Our findings indicate that active detection recruits left‐lateralized frontal and motor regions to support goal‐directed evaluation and action, whereas passive detection prioritizes right‐lateralized salience networks to support automatic reorienting. These divergences may reflect evolutionary trade‐offs: active detection optimizes precision, while passive detection prioritizes speed.

Passive conditions showed relatively greater convergence in left STG, right hippocampus, left PHG, and right MFG/IFG, as well as the insula. STG, particularly its transverse portion, plays a central role in auditory deviance detection (Strobel et al. 2008), consistent with the prevalence of auditory oddball paradigms among passive tasks and with automatic responses to perceptual mismatches. MTG/IFG convergence overlaps with regions of the ventral attention network implicated in stimulus‐driven reorienting (Corbetta et al. 2008), supporting the interpretation that passive paradigms rely heavily on bottom‐up mechanisms. The involvement of the right hippocampus and left PHG suggests that even without explicit memory demands, deviant events engage MTL regions to encode contextual or statistical irregularities, in line with evidence that hippocampal circuitry tracks statistical structure and detects violations during passive exposure (Kumaran and Maguire 2006, 2007). The recruitment of the insula, a core node of the salience network (Seeley et al. 2007), further underscores the reliance of passive detection on interoceptive and autonomic signaling to prioritize unexpected events.

Active conditions, in contrast, showed stronger convergence in the left precentral gyrus, left IFG, and dACC. The left IFG, particularly pars orbitalis and pars opercularis, has been linked to controlled retrieval, selection among competing representations, and response inhibition (Brass et al. 2005; Aron et al. 2014; Badre and Wagner 2007). In active novelty paradigms, these functions likely support the deliberate retrieval of stored information to evaluate stimulus familiarity, while suppressing automatic responses to allow for careful assessment. Left precentral gyrus, a hub for motor planning, is well positioned to support explicit behavioral responses (e.g., button presses) to novel targets (Nachev et al. 2008). Furthermore, the dACC is a region central to conflict monitoring and error detection (Botvinick et al. 2001). This pattern is consistent with active novelty detection's dependence on prediction error signaling: dACC compares incoming stimuli against expectations and, when mismatches are detected, engages prefrontal and hippocampal networks to update memory schemas and behavioral strategies (Alexander and Brown 2011). Coactivation of dACC with parahippocampal regions in active tasks further supports integration between mismatch detection and associative memory during explicit novelty judgements.

While both active and passive conditions engaged medial frontal regions, their functional contributions likely differ according to task demands. In active tasks, dACC and SMA appear more involved in cognitive control and action preparation, whereas in passive paradigms, the relative emphasis shifts toward insula and temporal cortices, highlighting sensory salience and automatic orienting. This dissociation mirrors our meta‐analytic contrasts: active detection's stronger convergence in SMA and precentral regions reflects motor planning and action selection, whereas passive detection's stronger convergence in insula and STG emphasizes sensory and salience‐driven aspects of novelty processing.

### 4.4 Limitations and Future Directions

Despite the valuable insights gained from this meta‐analysis, several methodological and conceptual limitations must be acknowledged.

A key challenge in this study was the inherent variability in experimental designs across fMRI studies. Our ALE approach was deliberately designed to integrate spatial convergence of reported coordinates across a wide range of paradigms in order to identify robust, task‐general regularities in novelty processing. While we made efforts to systematically classify tasks into active and passive novelty paradigms, inevitable variability in stimulus modality, task structure, and cognitive engagement levels could not be entirely controlled. Notably, some active tasks may have contained implicit cognitive demands, such as attentional shifts or salience‐driven reorienting, leading to potential overlaps in activation patterns between active and passive conditions. Furthermore, differences in stimulus type (e.g., visual vs. auditory stimuli) could have influenced activation profiles, particularly in modality‐specific sensory cortices. These factors may blur fine‐grained distinctions between active and passive conditions, but they also ensure that the networks identified here reflect effects that generalize across paradigms.

A second limitation concerns the broader theoretical challenge of differentiating true novelty processing from expectation violation or surprise (Barto et al. 2013; Modirshanechi et al. 2023; Strange et al. 2005). The hippocampus, a core node in our common network, is also central to predictive coding models in which it generates expectations about future input and signals prediction errors when those expectations are violated (Pezzulo et al. 2017; Monosov 2024; Zhang et al. 2022). Standard “novelty” paradigms, particularly oddball and mismatch designs, instantiate low‐probability events against a stable background, thereby confounding stimulus novelty, expectancy violation, and salience. Likewise, many “novel” stimuli in encoding tasks are both unfamiliar and contextually unexpected. As a result, our meta‐analytic contrasts cannot determine whether medial temporal convergence primarily reflects novelty per se or more general mismatch and surprise responses; they are best interpreted as revealing how task demands modulate the processing of events that are both novel and surprising. Progress on this issue will require paradigms that orthogonalize prior experience (new vs. familiar), predictive context (expected vs. unexpected), and task relevance, combined with computational modeling of novelty‐ and surprise‐related signals (Modirshanechi et al. 2023; Monosov 2024). In line with this, a recent primate electrophysiology study demonstrated that novelty, recency, and different forms of sensory surprise are encoded by overlapping but nonidentical neuronal populations across anterior ventral temporal cortex, basal forebrain, amygdala, striatum, and hippocampus (Zhang et al. 2022). These findings reinforce the need for human studies that treat novelty, recency, and surprise as related but partially dissociable dimensions.

A related limitation is the heterogeneity of novelty types across studies. As highlighted by recent work, stimulus (or absolute) novelty—encountering stimuli without pre‐existing memory representations—can be distinguished from contextual or relational novelty, in which familiar stimuli violate expectations in a given context (Quent et al. 2021; Schomaker et al. 2021; Schomaker and Meeter 2015). Our corpus included both types of paradigms; however, the number of studies was too small to permit a reliable and systematic coding that would support subgroup meta‐analyses. Consequently, the networks identified here likely blend processes related to unfamiliarity with those related to violations of learned regularities. At the same time, emerging fMRI studies have begun to implement designs that more cleanly target stimulus versus contextual novelty. For example, Harı and Demiralp (2025) used trial‐unique pseudo‐object images in a paradigm specifically intended to minimize contextual deviance and task relevance, finding robust responses in higher‐order visual regions (including fusiform and lateral occipital cortices) together with comparatively weaker effects in inferior parietal and supramarginal areas, consistent with a profile in which novelty is expressed predominantly in advanced sensory cortices when contextual demands are reduced. Other studies have manipulated contextual novelty more directly by varying whether familiar stimuli violate learned associations or expectations, showing that such violations recruit hippocampal–midbrain–striatal and parietal networks even when stimulus identity is held constant (Kafkas and Montaldi 2015). Together, these paradigms illustrate how stimulus and contextual novelty can be probed with more targeted designs, providing a foundation for future neuroimaging meta‐analyses that treat these dimensions as distinct subgroups rather than collapsing across them, as in the present work.

Finally, although we contrasted active and passive task demands, our findings are more consistent with a continuum than with two discrete systems. Passive, stimulus‐driven responses to deviant events can transition into active, goal‐directed evaluation when those events acquire behavioral relevance. Evidence from electroencephalography (EEG) studies supports this view, showing that the P3 novelty response can be dissociated into an earlier P3a component related to attentional orienting and a later P3b component related to task‐relevant evaluation (Osorio et al. 2022; Polich 2007; Ranganath and Rainer 2003). Building on our current results, we speculate that regions such as the IFG, previously proposed as a dynamic switch within the dorsal attention network, may play a pivotal role in mediating this transition by integrating mismatch signals with current goals. Future research using time‐resolved neuroimaging and connectivity analyses will be important to characterize how the novelty network reconfigures as processing shifts from passive detection to active evaluation and to clarify how stimulus‐driven and goal‐oriented streams are integrated to prioritize behaviorally relevant information.

## 5 Conclusion

To conclude, the present study employed a coordinate‐based ALE meta‐analysis to identify core similarities and differences between the brain networks subserving active and passive novelty processing. Our results reveal an integrative network in which deviant events consistently engage MTL structures and frontal salience/control regions, supporting memory‐based mismatch computations, salience registration, and preparation of behavioral responses. Within this shared backbone, active conditions are associated with stronger convergence in left‐lateralized frontal and motor regions, reflecting goal‐directed evaluation and response selection, whereas passive conditions show stronger convergence in right‐lateralized sensory and salience‐related areas, consistent with automatic attentional reorienting. By integrating active and passive paradigms into a unified quantitative and connectivity framework, this work bridges previously separate literatures and provides an integrative account of how task demands influence the neural architecture of novelty processing.

## Author Contributions

E.W. conceptualized the study and contributed to investigation, methodology, validation, software, formal analysis, data curation, visualization, project administration, and writing (original draft and review/editing). G.S. contributed to the investigation and writing (review/editing). I.S.R. contributed to investigation, visualization, and writing (review/editing). J.M. contributed to supervision and writing (review/editing). P.P. contributed to funding acquisition and writing (review/editing). All authors reviewed and approved the final manuscript.

## Supporting information




**Supplementary Materials**: brb371221‐sup‐0001‐SuppMat.docx

## Data Availability

Data will be made available upon reasonable request.
