# Supplementary material for: The Neural Blueprint of Novelty: A Meta‐Analytic Dissection of Active and Passive Novelty Processing Networks
Source: Brain Behav. 2026 Feb 9;16(2):e71221. doi: 10.1002/brb3.71221 (PMC12887445; doi:10.1002/brb3.71221)
Supplement: Supplementary file 1 — Supplementary Materials: brb371221‐sup‐0001‐SuppMat.docx [file BRB3-16-e71221-s001.docx]

| **Supplementary Table 1. Characteristics of the included studies categorized under active and passive paradigms.** | | | | | | | | | | |
| --- | --- | --- | --- | --- | --- | --- | --- | --- | --- | --- |
| Authors | Gender Ratio (M:F) | | Age | Modality | Task Paradigm | Stimuli Type | Contrasts | Coordinates | Number of Foci | Location |
| ***Active*** | |  |  |  |  |  |  |  |  |  |
| Barman et al. (2014) | | 119 (61:58) | M = 24.35; SD = 2.60 | Vision | Incidental Encoding Task | Novel and Familiar Indoor/Outdoor Pictures | Novel > Familiar | MNI | 20 | Table S6 |
| Bowman et al. (2015) | | 17 (6:11) | M = 21.28; SD = 1.79 | Vision | Old-New Judgement | 1092 color photographs of common objects from Internet search | Correct rejections > False recognition | TAL | 2 | Table S2 |
| Braskie et al. (2012) | | 26 (4:22) | M = 31.6 | Vision | Novelty Encoding Task | Pictures | Novel > Repeated | MNI | 21 | Table 2 |
| Bubic et al. (2011) | | 25 (25) | M = 26.7 | Vision | Serial Prediction Task | Patterns | Violated vs. ordered object sequence | TAL | 3 | Table 1 |
|  | |  |  | Vision | Serial Prediction Task | Patterns | Violated vs. ordered position sequence | TAL | 7 | Table 1 |
|  | |  |  | Vision | Serial Prediction Task | Patterns | Violated vs. ordered rhythm sequence | TAL | 4 | Table 1 |
| Bunzeck et al. (2012) | | 14 (5:9) | M = 22.4; SD = 3.8 | Vision | Recognition memory based preference judgment task | Scenes | Main Novelty | MNI | 20 | Table S1A |
| Daselaar et al. (2006) | | 14 (8:6) | M = 21.7; SD = 2.4 | Vision | Old-New Judgement | 240 five-letter words selected from the MRC psycholinguistic database | Novelty Parametric | MNI | 13 | Table 2 |
| de Chastelaine et al. (2017) | | 136 (65:71) | 18-76 | Vision | Old-New Judgement | 320 semantically-unrelated word pairs randomly divided into four lists of 80 pairs | Correctly rejected > associative hits | MNI | 9 | Table 4 |
| Dudukovic et al. (2007) | | 18 (9:9) | M = 20.6 | Visual | Old-New Judgment | 396 abstract and concrete nouns matched for word length and frequency | Correct Novelty > Correct Recency | MNI | 9 | Table 4 |
| Düzel et al. (2004) | | 11 (4:7) | M = 24.6; SD = 2.6 | Visual | Old-New Judgment | Location of object | Novel Locations > Old Locations | MNI | 3 | In Text |
| Evans et al. (2017) | | 40 (19:21) | 18-28 | Vision | Old-New Judgment | 180 "new" and "old" words | New words > Old words | MNI | 4 | Table 7 |
| Geiger et al. (2018) | | 74 (31:43) | M = 26; SD = 7.0 | Vision | Steinberg Item Recognition Task | Target and probe letters | Novel Stimuli > Practiced Stimuli | MNI | 26 | Table 1 |
| Herweg et al. (2018) | | 21 (NA) | 22-34 | Vision | Old-New Judgement | 160 outdoor and indoor scenes | New > Old scenes | MNI | 7 | Table 2 |
| Kafkas & Montaldi (2014) | | 17 (7:10) | M = 22.6; SD = 4.2 | Vision | Familiarity and Novelty Rating Task | Pictures depicting man-made and natural objects | Monotonic increase with Novelty | MNI | 12 | Table 2 |
| Kaplan et al. (2014) | | 20 (20:0) | M = 23.9; SD = 3.7 | Vision | Object-Location Memory Task | Scenes | Main Effect of Environment Novelty | MNI | 7 | Table 3 |
|  | |  |  | Vision | Object-Location Memory Task | Object | Main Effect of Object Novelty | MNI | 5 | Table 4 |
| Kim et al. (2010) | | 12 (6:6) | 18-31 | Vision | Memory Encoding and Recognition | 72 categorical 6-word lists selected from natural or artificial category norms | High-confidence correct rejection > high-confidence hit | TAL | 7 | Table 4D |
| Krebs et al. (2009)* | | 24 (12:12) | M = 23; SD = 1.7 | Vision | Delayed Monetary Incentive Task | Indoor or outdoor scenes | Experiment 1 (Novel > Familiar) | MNI | 9 | Table S2 |
| Krebs et al. (2013) | | 18 (6:12) | M = 22.5 | Vision | Picture-word Interference Task | Indoor or outdoor scenes | Novel Pictures > Familiar Pictures | MNI | 4 | Table 2 |
| Lawson et al. (2012) | | 40 (20:20) | 18-25 | Vision | Old-New Task | 280 two-dimensional pictures of common objects | New > Old | MNI | 9 | Table 1 |
| Manelis et al. (2017) | | 33 (NA) | M = 22.3 | Vision | Modified Delayed-Match-To-Sample Task | 136 pictures of everyday items | New > Old | MNI | 6 | Table 2 |
|  | |  |  | Vision | Modified Delayed-Match-To-Sample Task | 136 pictures of everyday items | New > Pseudo new | MNI | 6 | Table 2 |
| Plailly et al. (2007) | | 13 (13) | M = 24.54; SD = 2.44 | Olfaction | Familiarity Judgement Task | Forty-eight odorants a priori determined as familiar and unfamiliar | Olfaction (Unfamiliar > Familiar) | MNI | 3 | Table 2 |
|  | |  |  | Auditory | Familiarity Judgement Task | Forty-eight music excerpts a priori determined as familiar and unfamiliar | Music (Unfamiliar > Familiar) | MNI | 5 | Table 2 |
| Poppenk et al. (2010) | | 13 (NA) | M = 25.8 | Vision | Encoding-Recognition Task | 384 scenes selected from a set of photographs depicting emotionally neutral object configurations, rooms and landscapes | Novel > Familiar | MNI | 5 | Table S1 |
| Schott B et al. (2023) Cohort 1 | | 117 (60:57) | M = 24.37; SD = 2.60 | Vision | Old-New Recognition Task | Indoor and outdoor scenes | Novel > master images | MNI | 26 | Table S2 |
| Schott B et al. (2023) Cohort 2 | | 58 (30:28) | M = 23.62; SD = 3.45 | Vision | Old-New Recognition Task | Scenes | Novel > master images | MNI | 11 | Table S3 |
| Schott B et al. (2023) Cohort 3 | | 64 (27:37) | M = 24.45; SD = 4.46 | Vision | Old-New Recognition Task | Scenes | Novel > master images | MNI | 20 | Table S4 |
| Schwartz et al. (2003) | | 8 (4:4) | M = 22.4; SD = 1.3 | Vision | Old-New Recognition Task | Neutral Faces | Novel > Familiar Neutral Faces | TAL | 6 | Table 1 |
| Wittmann et al. (2007) | | 15 (7:8) | M = 24.5; SD = 4.0 | Visual | predictive familiarity and novelty categorization task | Coloured Cue indicating Novel or Familiar Picture | Novel Anticipation Response | TAL | 31 | Table 3 |
| Zäske et al. (2017) | | 24 (12:12) | M = 21.6 | Auditory | Old-New Judgement Tasl | Recordings from 60 adult native speakers of German (speaker Identity) | Voice Novelty Effect | MNI | 4 | Table 2 |
|  | |  |  |  |  |  |  |  |  |  |
| ***Passive*** | |  |  |  |  |  |  |  |  |  |
| Balderston et al. (2011) Experiment 1 Sample | | 20 (6:14) | M = 20.78; SD = 2.90 | Vision | fMRI Adaption Paradigm | Emotional (mutilated bodies) and Neutral (Healthy individuals with gaze directed toward the camera) Images of human from the International Affective Picture System database | Novel > Repeat | TAL | 9 | Supp Table 1 |
| Balderston et al. (2011) Experiment 2 Sample | | 20 (8:12) | M = 20.78; SD = 2.90 | Vision | fMRI Adaption Paradigm | Emotional (mutilated bodies) and Neutral (Healthy individuals with gaze directed toward the camera) Images of human from the International Affective Picture System database | Novel > Repeat | TAL | 6 | Supp Table 2 |
| Balderston et al. (2013) | | 18 (NA) | M = 21.72; SD = 3.56 | Vision | fMRI Adaption Paradigm | Images of Snakes and Flowers from the International Affective Picture System Database | Novel > Repeat | TAL | 14 | Table 2 |
| Ballard et al. (2017) | | 27 (13:14) | M = 23.3; SD = 8.6 | Taste | Habituation Paradigm | volution brand vegetable juices | Habituation Contrast | TAL | 12 | Table 1 |
| Binder et al. (2005) | | 32 (16:16) | 18-35 | Vision | Novel Scrambled task | Novel/Repeating Pictures of Indoor/Outdoor Scenes | Novel Pictures > Repeated Pictures | TAL | 15 | Table 2 |
| Blackford et al. (2010) | | 29 (NA) | M = 22; SD = 3.1 | Vision | Passive viewing | Common Pictures from the International Affective Picture Set, and Novel Uncommon Pictures from Public Domain | Novel Common > Familiar Stimuli | MNI | 8 | Table S2 |
|  | |  |  | Vision | Passive viewing | Common Pictures from the International Affective Picture Set, and Novel Uncommon Pictures from Public Domain | Novel Uncommon > Novel Common | MNI | 9 | Table S3 |
| Cacciaglia et al. (2015) | | 12 (5:7) | M = 27.5; SD = 3.4 | Auditory | passive frequency oddball paradigm | Tones | Deviant > Standard | MNI | 2 | In Text S3.1 |
| Cacciaglia et al. (2019) | | 15 (6:9) | M = 25.6; SD = 4.3 | Auditory | passive frequency oddball paradigm | Tones | Deviant > Standard | MNI | 3 | In Text S3.1 |
| Collier et al. (2014) | | 21 (NA) | NA | Auditory | Visual + Auditory Oddball | Tones | Auditory Novel > Auditory Standard | MNI | 21 | SuppMat |
|  | |  |  | Vision | Visual + Auditory Oddball | Pictures | Vision Novel > Vision Standard | MNI | 17 | SuppMat |
| Domínguez-Borrs et al. (2009) | | 17 (0:17) | M = 22, SD = 3.35 | Auditory | auditory–visual distraction paradigm | Valanced faces paired with valanced audio | Novel Neutral > Standard Neutral | TAL | 21 | Table 2 |
|  | |  |  | Auditory | auditory–visual distraction paradigm | Valanced faces paired with valanced audio | Novel Negative > Standard Negative | TAL | 8 | Table 2 |
| Downar et al. (2002) | | 10 (6:4) | 23-46 | VisionAuditory/Tactile | Multimodal Passive Novelty Oddball | Multimodal Sequences interspaced with Novel Stimulation | Conjunction of Novel > Familiar across Visual, Auditory and Tactile | TAL | 7 | Table 1 |
| Fenker et al. (2008) | | 32 (12:20) | 20-35 | Vision | Incidental Encoding Task | 300 neutral German words of 4–10 letters denoting living and non-living concepts | Novel Images > Familiar images | MNI | 13 | Table 4 |
| Fischer et al. (2003) | | 8 (8:0) | M = 25.5; SD = 6.1 | Vision | Repetition Suppression | Face stimuli consisting of PICT files displaying two male and two female individuals showing both fearful and neutral expressions | Repetition Suppression | TAL | 4 | Table 1 |
| Friedman et al. (2009) | | 15 (15:0) | M = 25 | Auditory | Oddball | Pure tones at 500-350 Hz of 336 ms and 48 unique environmental sounds described by Fabiani et al. [1996](https://pmc.ncbi.nlm.nih.gov/articles/PMC2718677/#bib6) chosen from six categories (animal, bird, human, musical instrument, environmental, and electronic) of 159-399 ms | Novel > Baseline | MNI | 10 | Table 2 |
| Greene et al. (2015) | | 19 (11:8) | 18-30 | Visual | Delayed Match to Sample Task | Abstract monochrome shapes | Novel Trials > WM Trials | MNI | 1 | Table 1 |
| Guitart-Masip et al. (2010) | | 16 (7:9) | M = 23.8; SD = 3.84 | Vision | Decision Making | Ten outdoor/indoor scenes and three fractal images | Novel > Familiar | MNI | 72 | Table S2 |
| Gur et al.(2007) | | 36 (17:19) | M = 30.1; SD = 8.3 | Vision | Visual Oddball | Circular arrangement of small bright red (Standard) or green (Target) Gabor elements, and fractal images (Novel) | Novel > Baseline | TAL | 14 | Table 1 |
| Hawco et al. (2014) | | 22 (NA) | 18-35 | Vision | Repetition Suppression | Triads of common objects | Stimulus Novelty (novel > repeat) | MNI | 14 | Table 1 |
| Howard et al. (2011) | | 20 (10:10) | M = 25.3; SD = 3.9 | Vision | target detection task | 289 combinations of animate and non-animate objects background indoor and outdoor scenes obtained from the Hemera Photo-Objects image collection or other sources | Novel Scene > Repeated Scene | MNI | 4 | Table 1 |
| Huang et al. (2012) | | 18 (7:11) | 19-28 | Audio | Cued Auditory Attention Shifting Task | Brief pure tones of 50 ms with 5-ms ramps presented randomly to the right (800 Hz) or left ear (1500 Hz) preceded by a 250-ms buzzer cue sound | (Cue + Novel + Standard) > (Cue + Standard) | MNI | 64 | Table 2 |
| Jessen et al. (2002) | | 8 (5:3) | M = 24; SD = 1.7 | Vision | Passive Viewing Task | 100 novel and repeated pictures of complex indoor and outdoor scenes including objects and animals | Novel > Repeated | MNI | 10 | Table 1 |
| Krebs et al. (2009)* | | 20 (10:10) | M = 25; SD = 2.9 | Vision | Delayed Monetary Incentive Task | Indoor or outdoor scenes | Experiment 2 (Novel > Familiar) | MNI | 12 | Table S6 |
| Kumaran et al. (2006) | | 17 (8:9) | M = 26 | Vision | speed target-detection task. | Quartets of objects from the Hemera Photo-Objects image collection placed on a white background | First > Baseline | MNI | 8 | Table S1 |
| Laurens et al. (2005) | | 28 (21:7) | M = 28.2; SD = 8.9 | Auditory | Auditory Oddball | Auditory stimuli including repeating target stimuli (1500 Hz tones), novel stimuli (non-repeating digital noises), and repeating non-target stimuli (1000 Hz tones) | Novel > Non Target Stimulus | TAL | 29 | Table 2 |
| Modinos et al. (2020) | | 31 (15:16) | M = 25.0; SD = 4.1 | Vision | Visual Oddball | Black-and-white outdoor scenes | Novel > Neutral Oddballs | MNI | 9 | eTable 4 |
| Murty et al. (2013) | | 24 (11:13) | M= 25 | Vision | Visual Oddball | Scenes | Novel > Familiar | MNI | 2 | In Text |
| Opitz et al. (1999) | | 14 (NA) | M = 22 | Auditory | Auditory Oddball | Pure sine tones at 600 and 1000 Hz and unique environmental sounds (novels) | Unattended Condition (Novel Stimuli) | TAL | 4 | Table 1 |
|  | |  |  | Auditory | Auditory Oddball | Pure sine tones at 600 and 1000 Hz and unique environmental sounds (novels) | Attended Condition (Novel Stimuli) | TAL | 4 | Table 2 |
| Kiehl et al. (2001) | | 10 (5:5) | M = 26.3; SD = 7.2 | Auditory | Auditory Oddball | Tones | Novel Stimuli > Nontarget Baseline Condition | TAL | 24 | Table 2 |
|  | |  |  | Auditory | Auditory Oddball | Tones | Novel > Target Stimuli | TAL | 27 | Table 4 |
| Pihlajamäki et al. (2005) | | 12 (6:6) | 21-29 | Vision | Visuospatial fMRI Activation Task | Grids with objects | (Novel Object > Baseline) Conj. (Novel Location > Baseline) | TAL | 11 | Table 4 |
| Stoppel et al. (2009) | | 18 (6:12) | M = 24.6; SD = 3.2 | Vision | Visual selective attention task | Fractal Pictures | Attended novels > Attended standards | MNI | 7 | Table 2 |
|  | |  |  | Vision | Visual selective attention task | Fractal Pictures | Unattended Novels | MNI | 6 | Table 2 |
| Tamminga et al. (2012) | | 18 (5:13) | M = 40.3; SD = 11.2 | Vision | Incidental Encoding Task | Novel/Familiar Scenes | Novelty Sensitive Regions | MNI | 32 | Table S1 |
| Tegelbeckers et al. (2015) | | 19 (19:0) | M = 13.58 | Vision | Visual Oddball | Black‐and‐white pictures of landscape scenes | Novel > Standard | MNI | 7 | Table 2 |
| Tegelbeckers et al. (2022) | | 25 (25:0) | M = 13.6 | Vision/Auditory | Flanker Task with Sound Cues | Novel Standard Sound distractors with flanker task | Novel > No sound | MNI | 2 | Table 2 |
|  | |  |  | Vision/Auditory | Flanker Task with Sound Cues | Novel Standard Sound distractors with flanker task | Novel > Standard Sounds | MNI | 3 | Table 2 |
| Weierich et al. (2010) | | 15 (7:8) | M = 22.2; SD = 2.37 | Vision | Arousal Rating Task | images from the International Affective Picture System | Novel > Familiar | TAL | 31 | Table 1 |
| Wessel et al. (2012) | | 17 (NA) | M = 24.7; SD = 2.6 | Vision | Flanker + Visual Oddball Task | Triangles and Novel Stimuli from the International Picture Naming Project | Novel > Standard | MNI | 47 | Table 2 |
| Winton-Brown et al. (2017) | | 32 (18:14) | M = 23.69; SD = 4.08 | Vision | Salience Integration Task | Scenes from the International Affective Picture System in a Salience Integration Task | Novelty Effects | MNI | 11 | Table S1B |
| Witt et al. (2010) | | 33 (22:11) | M = 33.2; SD = 13.8 | Auditory | Passive Auditory Oddball | Novel, standard and target tones | Novelty detection | MNI | 5 | Table 2 |
| Wolf et al. (2008) | | 21 (11:10) | M = 28.6; SD = 7.1 | Auditory | Passive Auditory Oddball | Frequent, Infrequent and Novel Tones | Novel > Baseline | MNI | 22 | Table 3 |
| Yamaguchi et al. (2004) | | 10 (1:9) | M = 19.3; SD = 1.6) | Vision | Passive Visual Oddball with Attended and Unattended conditions | Standard, Target and Novel coloured images | Attended Novel Stimuli > Standard | TAL | 16 | Table 1 |
|  | |  |  | Vision | Passive Visual Oddball with Attended and Unattended conditions | Standard, Target and Novel coloured images | Unattended Novel Stimuli > Standard | TAL | 7 | Table 1 |

**References**

Balderston, N. L., Schultz, D. H., & Helmstetter, F. J. (2011). The human amygdala plays a stimulus specific role in the detection of novelty. *NeuroImage*, *55*(4), 1889–1898. <https://doi.org/10.1016/j.neuroimage.2011.01.034>

Balderston, N. L., Schultz, D. H., & Helmstetter, F. J. (2013). The Effect of Threat on Novelty Evoked Amygdala Responses. *PLoS ONE*, *8*(5), e63220. <https://doi.org/10.1371/journal.pone.0063220>

Ballard, I. C., Hennigan, K., & McClure, S. M. (2017). Mere Exposure: Preference Change for Novel Drinks Reflected in Human Ventral Tegmental Area. *Journal of Cognitive Neuroscience*, *29*(5), 793–804. <https://doi.org/10.1162/jocn_a_01098>

Barman, A., Assmann, A., Richter, S., Soch, J., SchÃ¼tze, H., WÃ¼stenberg, T., Deibele, A., Klein, M., Richter, A., Behnisch, G., DÃ¼zel, E., Zenker, M., Seidenbecher, C. I., & Schott, B. H. (2014). Genetic variation of the RASGRF1 regulatory region affects human hippocampus-dependent memory. *Frontiers in Human Neuroscience*, *8*. <https://doi.org/10.3389/fnhum.2014.00260>

Binder, J. R., Bellgowan, P. S. F., Hammeke, T. A., Possing, E. T., & Frost, J. A. (2005). A Comparison of Two *f* MRI Protocols for Eliciting Hippocampal Activation. *Epilepsia*, *46*(7), 1061–1070. <https://doi.org/10.1111/j.1528-1167.2005.62004.x>

Blackford, J. U., Buckholtz, J. W., Avery, S. N., & Zald, D. H. (2010). A unique role for the human amygdala in novelty detection. *NeuroImage*, *50*(3), 1188–1193. <https://doi.org/10.1016/j.neuroimage.2009.12.083>

Bowman, C. R., & Dennis, N. A. (2015). The neural correlates of correctly rejecting lures during memory retrieval: The role of item relatedness. *Experimental Brain Research*, *233*(6), 1963–1975. <https://doi.org/10.1007/s00221-015-4268-y>

Braskie, M. N., Medina, L. D., Rodriguez-Agudelo, Y., Geschwind, D. H., Macias-Islas, M. A., Cummings, J. L., Bookheimer, S. Y., & Ringman, J. M. (2012). Increased fMRI signal with age in familial Alzheimer’s disease mutation carriers. *Neurobiology of Aging*, *33*(2), 424.e11-424.e21. <https://doi.org/10.1016/j.neurobiolaging.2010.09.028>

Bubic, A., Von Cramon, D. Y., & Schubotz, R. I. (2011). Exploring the detection of associatively novel events using fMRI. *Human Brain Mapping*, *32*(3), 370–381. <https://doi.org/10.1002/hbm.21027>

Bunzeck, N., Doeller, C. F., Dolan, R. J., & Duzel, E. (2012). Contextual interaction between novelty and reward processing within the mesolimbic system. *Human Brain Mapping*, *33*(6), 1309–1324. <https://doi.org/10.1002/hbm.21288>

Cacciaglia, R., Costa-Faidella, J., Zarnowiec, K., Grimm, S., & Escera, C. (2019). Auditory predictions shape the neural responses to stimulus repetition and sensory change. *NeuroImage*, *186*, 200–210. <https://doi.org/10.1016/j.neuroimage.2018.11.007>

Cacciaglia, R., Escera, C., Slabu, L., Grimm, S., Sanjuán, A., Ventura-Campos, N., & Ávila, C. (2015). Involvement of the human midbrain and thalamus in auditory deviance detection. *Neuropsychologia*, *68*, 51–58. <https://doi.org/10.1016/j.neuropsychologia.2015.01.001>

Collier, A. K., Wolf, D. H., Valdez, J. N., Turetsky, B. I., Elliott, M. A., Gur, R. E., & Gur, R. C. (2014). Comparison of auditory and visual oddball fMRI in schizophrenia. *Schizophrenia Research*, *158*(1–3), 183–188. <https://doi.org/10.1016/j.schres.2014.06.019>

Daselaar, S. M., Fleck, M. S., & Cabeza, R. (2006). Triple Dissociation in the Medial Temporal Lobes: Recollection, Familiarity, and Novelty. *Journal of Neurophysiology*, *96*(4), 1902–1911. <https://doi.org/10.1152/jn.01029.2005>

De Chastelaine, M., Mattson, J. T., Wang, T. H., Donley, B. E., & Rugg, M. D. (2017). Independent contributions of fMRI familiarity and novelty effects to recognition memory and their stability across the adult lifespan. *NeuroImage*, *156*, 340–351. <https://doi.org/10.1016/j.neuroimage.2017.05.039>

Domínguez-Borràs, J., Trautmann, S.-A., Erhard, P., Fehr, T., Herrmann, M., & Escera, C. (2009). Emotional Context Enhances Auditory Novelty Processing in Superior Temporal Gyrus. *Cerebral Cortex*, *19*(7), 1521–1529. <https://doi.org/10.1093/cercor/bhn188>

Downar, J., Crawley, A. P., Mikulis, D. J., & Davis, K. D. (2002). A Cortical Network Sensitive to Stimulus Salience in a Neutral Behavioral Context Across Multiple Sensory Modalities. *Journal of Neurophysiology*, *87*(1), 615–620. <https://doi.org/10.1152/jn.00636.2001>

Dudukovic, N. M., & Wagner, A. D. (2007). Goal-dependent modulation of declarative memory: Neural correlates of temporal recency decisions and novelty detection. *Neuropsychologia*, *45*(11), 2608–2620. <https://doi.org/10.1016/j.neuropsychologia.2007.02.025>

Düzel, E., Habib, R., Guderian, S., & Heinze, H. J. (2004). Four types of novelty–familiarity responses in associative recognition memory of humans. *European Journal of Neuroscience*, *19*(5), 1408–1416. <https://doi.org/10.1111/j.1460-9568.2004.03253.x>

Evans, S., Dowell, N. G., Tabet, N., King, S. L., Hutton, S. B., & Rusted, J. M. (2017). Disrupted neural activity patterns to novelty and effort in young adult *APOE* ‐e4 carriers performing a subsequent memory task. *Brain and Behavior*, *7*(2), e00612. <https://doi.org/10.1002/brb3.612>

Fenker, D. B., Frey, J. U., Schuetze, H., Heipertz, D., Heinze, H.-J., & Duzel, E. (2008). Novel Scenes Improve Recollection and Recall of Words. *Journal of Cognitive Neuroscience*, *20*(7), 1250–1265. <https://doi.org/10.1162/jocn.2008.20086>

Fischer, H., Wright, C. I., Whalen, P. J., McInerney, S. C., Shin, L. M., & Rauch, S. L. (2003). Brain habituation during repeated exposure to fearful and neutral faces: A functional MRI study. *Brain Research Bulletin*, *59*(5), 387–392. <https://doi.org/10.1016/S0361-9230(02)00940-1>

Friedman, D., Goldman, R., Stern, Y., & Brown, T. R. (2009). The brain’s orienting response: An event‐related functional magnetic resonance imaging investigation. *Human Brain Mapping*, *30*(4), 1144–1154. <https://doi.org/10.1002/hbm.20587>

Geiger, L. S., Moessnang, C., Schäfer, A., Zang, Z., Zangl, M., Cao, H., Van Raalten, T. R., Meyer-Lindenberg, A., & Tost, H. (2018). Novelty modulates human striatal activation and prefrontal–striatal effective connectivity during working memory encoding. *Brain Structure and Function*, *223*(7), 3121–3132. <https://doi.org/10.1007/s00429-018-1679-0>

Greene, C. M., Vidaki, K., & Soto, D. (2015). Fractionating the neural substrates of incidental recognition memory. *Learning & Memory*, *22*(1), 24–30. <https://doi.org/10.1101/lm.036327.114>

Guitart-Masip, M., Bunzeck, N., Stephan, K. E., Dolan, R. J., & Düzel, E. (2010). Contextual Novelty Changes Reward Representations in the Striatum. *The Journal of Neuroscience*, *30*(5), 1721–1726. <https://doi.org/10.1523/JNEUROSCI.5331-09.2010>

Gur, R. C., Turetsky, B. I., Loughead, J., Waxman, J., Snyder, W., Ragland, J. D., Elliott, M. A., Bilker, W. B., Arnold, S. E., & Gur, R. E. (2007). Hemodynamic responses in neural circuitries for detection of visual target and novelty: An event‐related fMRI study. *Human Brain Mapping*, *28*(4), 263–274. <https://doi.org/10.1002/hbm.20319>

Hawco, C., & Lepage, M. (2014). Overlapping patterns of neural activity for different forms of novelty in fMRI. *Frontiers in Human Neuroscience*, *8*. <https://doi.org/10.3389/fnhum.2014.00699>

Herweg, N. A., Sommer, T., & Bunzeck, N. (2018). Retrieval Demands Adaptively Change Striatal Old/New Signals and Boost Subsequent Long-Term Memory. *The Journal of Neuroscience*, *38*(3), 745–754. <https://doi.org/10.1523/JNEUROSCI.1315-17.2017>

Howard, L. R., Kumaran, D., Ólafsdóttir, H. F., & Spiers, H. J. (2011). Double Dissociation between Hippocampal and Parahippocampal Responses to Object–Background Context and Scene Novelty. *The Journal of Neuroscience*, *31*(14), 5253–5261. <https://doi.org/10.1523/JNEUROSCI.6055-10.2011>

Huang, S., Belliveau, J. W., Tengshe, C., & Ahveninen, J. (2012). Brain Networks of Novelty-Driven Involuntary and Cued Voluntary Auditory Attention Shifting. *PLoS ONE*, *7*(8), e44062. <https://doi.org/10.1371/journal.pone.0044062>

Jessen, F., Manka, C., Scheef, L., Granath, D., Schild, H. H., & Heun, R. (2002). Novelty detection and repetition suppression in a passive picture viewing task: A possible approach for the evaluation of neuropsychiatric disorders. *Human Brain Mapping*, *17*(4), 230–236. <https://doi.org/10.1002/hbm.10071>

Kafkas, A., & Montaldi, D. (2014). Two separate, but interacting, neural systems for familiarity and novelty detection: A dual‐route mechanism. *Hippocampus*, *24*(5), 516–527. <https://doi.org/10.1002/hipo.22241>

Kaplan, R., Horner, A. J., Bandettini, P. A., Doeller, C. F., & Burgess, N. (2014). Human hippocampal processing of environmental novelty during spatial navigation. *Hippocampus*, *24*(7), 740–750. <https://doi.org/10.1002/hipo.22264>

Kiehl, K. A., Laurens, K. R., Duty, T. L., Forster, B. B., & Liddle, P. F. (2001). Neural sources involved in auditory target detection and novelty processing: An event-related fMRI study. *Psychophysiology*, *38*(1), 133–142. <https://doi.org/10.1017/S0048577201981867>

Kim, H., Daselaar, S. M., & Cabeza, R. (2010). Overlapping brain activity between episodic memory encoding and retrieval: Roles of the task-positive and task-negative networks. *NeuroImage*, *49*(1), 1045–1054. <https://doi.org/10.1016/j.neuroimage.2009.07.058>

Krebs, R. M., Fias, W., Achten, E., & Boehler, C. N. (2013). Picture novelty attenuates semantic interference and modulates concomitant neural activity in the anterior cingulate cortex and the locus coeruleus. *NeuroImage*, *74*, 179–187. <https://doi.org/10.1016/j.neuroimage.2013.02.027>

Krebs, R. M., Schott, B. H., Schütze, H., & Düzel, E. (2009). The novelty exploration bonus and its attentional modulation☆. *Neuropsychologia*, *47*(11), 2272–2281. <https://doi.org/10.1016/j.neuropsychologia.2009.01.015>

Kumaran, D., & Maguire, E. A. (2006). An Unexpected Sequence of Events: Mismatch Detection in the Human Hippocampus. *PLoS Biology*, *4*(12), e424. <https://doi.org/10.1371/journal.pbio.0040424>

Laurens, K. R., Kiehl, K. A., Ngan, E. T. C., & Liddle, P. F. (2005). Attention orienting dysfunction during salient novel stimulus processing in schizophrenia. *Schizophrenia Research*, *75*(2–3), 159–171. <https://doi.org/10.1016/j.schres.2004.12.010>

Lawson, A. L., Liu, X., Joseph, J., Vagnini, V. L., Kelly, T. H., & Jiang, Y. (2012). Sensation seeking predicts brain responses in the old–new task: Converging multimodal neuroimaging evidence. *International Journal of Psychophysiology*, *84*(3), 260–269. <https://doi.org/10.1016/j.ijpsycho.2012.03.003>

Manelis, A., Popov, V., Paynter, C., Walsh, M., Wheeler, M. E., Vogt, K. M., & Reder, L. M. (2017). Cortical Networks Involved in Memory for Temporal Order. *Journal of Cognitive Neuroscience*, *29*(7), 1253–1266. <https://doi.org/10.1162/jocn_a_01123>

Modinos, G., Allen, P., Zugman, A., Dima, D., Azis, M., Samson, C., Bonoldi, I., Quinn, B., Gifford, G. W. G., Smart, S. E., Antoniades, M., Bossong, M. G., Broome, M. R., Perez, J., Howes, O. D., Stone, J. M., Grace, A. A., & McGuire, P. (2020). Neural Circuitry of Novelty Salience Processing in Psychosis Risk: Association With Clinical Outcome. *Schizophrenia Bulletin*, *46*(3), 670–679. <https://doi.org/10.1093/schbul/sbz089>

Murty, V. P., Ballard, I. C., Macduffie, K. E., Krebs, R. M., & Adcock, R. A. (2013). Hippocampal networks habituate as novelty accumulates. *Learning & Memory*, *20*(4), 229–235. <https://doi.org/10.1101/lm.029728.112>

Opitz, B. (1999). The Functional Neuroanatomy of Novelty Processing: Integrating ERP and fMRI Results. *Cerebral Cortex*, *9*(4), 379–391. <https://doi.org/10.1093/cercor/9.4.379>

Pihlajamäki, M., Tanila, H., Könönen, M., Hänninen, T., Aronen, H. J., & Soininen, H. (2005). Distinct and overlapping fMRI activation networks for processing of novel identities and locations of objects. *European Journal of Neuroscience*, *22*(8), 2095–2105. <https://doi.org/10.1111/j.1460-9568.2005.04380.x>

Plailly, J., Tillmann, B., & Royet, J.-P. (2007). The Feeling of Familiarity of Music and Odors: The Same Neural Signature? *Cerebral Cortex*, *17*(11), 2650–2658. <https://doi.org/10.1093/cercor/bhl173>

Poppenk, J., McIntosh, A. R., Craik, F. I. M., & Moscovitch, M. (2010). Past Experience Modulates the Neural Mechanisms of Episodic Memory Formation. *The Journal of Neuroscience*, *30*(13), 4707–4716. <https://doi.org/10.1523/JNEUROSCI.5466-09.2010>

Schott, B. H., Soch, J., Kizilirmak, J. M., Schütze, H., Assmann, A., Maass, A., Ziegler, G., Sauvage, M., & Richter, A. (2023). Inhibitory temporo-parietal effective connectivity is associated with explicit memory performance in older adults. *iScience*, *26*(10), 107765. <https://doi.org/10.1016/j.isci.2023.107765>

Schwartz, C. E., Wright, C. I., Shin, L. M., Kagan, J., Whalen, P. J., McMullin, K. G., & Rauch, S. L. (2003). Differential amygdalar response to novel versus newly familiar neutral faces: A functional MRI probe developed for studying inhibited temperament. *Biological Psychiatry*, *53*(10), 854–862. <https://doi.org/10.1016/S0006-3223(02)01906-6>

Stoppel, C. M., Boehler, C. N., Strumpf, H., Heinze, H., Hopf, J. M., Düzel, E., & Schoenfeld, M. A. (2009). Neural correlates of exemplar novelty processing under different spatial attention conditions. *Human Brain Mapping*, *30*(11), 3759–3771. <https://doi.org/10.1002/hbm.20804>

Tamminga, C. A., Thomas, B. P., Chin, R., Mihalakos, P., Youens, K., Wagner, A. D., & Preston, A. R. (2012). Hippocampal novelty activations in schizophrenia: Disease and medication effects. *Schizophrenia Research*, *138*(2–3), 157–163. <https://doi.org/10.1016/j.schres.2012.03.019>

Tegelbeckers, J., Brechmann, A., Breitling-Ziegler, C., Bonath, B., Flechtner, H.-H., & Krauel, K. (2022). Neural Mechanisms Underlying the Effects of Novel Sounds on Task Performance in Children With and Without ADHD. *Frontiers in Human Neuroscience*, *16*, 878994. <https://doi.org/10.3389/fnhum.2022.878994>

Tegelbeckers, J., Bunzeck, N., Duzel, E., Bonath, B., Flechtner, H., & Krauel, K. (2015). Altered salience processing in attention deficit hyperactivity disorder. *Human Brain Mapping*, *36*(6), 2049–2060. <https://doi.org/10.1002/hbm.22755>

Weierich, M. R., Wright, C. I., Negreira, A., Dickerson, B. C., & Barrett, L. F. (2010). Novelty as a dimension in the affective brain. *NeuroImage*, *49*(3), 2871–2878. <https://doi.org/10.1016/j.neuroimage.2009.09.047>

Wessel, J. R., Danielmeier, C., Morton, J. B., & Ullsperger, M. (2012). Surprise and Error: Common Neuronal Architecture for the Processing of Errors and Novelty. *The Journal of Neuroscience*, *32*(22), 7528–7537. <https://doi.org/10.1523/JNEUROSCI.6352-11.2012>

Winton-Brown, T., Schmidt, A., Roiser, J. P., Howes, O. D., Egerton, A., Fusar-Poli, P., Bunzeck, N., Grace, A. A., Duzel, E., Kapur, S., & McGuire, P. (2017). Altered activation and connectivity in a hippocampal–basal ganglia–midbrain circuit during salience processing in subjects at ultra high risk for psychosis. *Translational Psychiatry*, *7*(10), e1245–e1245. <https://doi.org/10.1038/tp.2017.174>

Witt, S. T., Lovejoy, D. W., Pearlson, G. D., & Stevens, M. C. (2010). Decreased prefrontal cortex activity in mild traumatic brain injury during performance of an auditory oddball task. *Brain Imaging and Behavior*, *4*(3–4), 232–247. <https://doi.org/10.1007/s11682-010-9102-3>

Wittmann, B. C., Bunzeck, N., Dolan, R. J., & Düzel, E. (2007). Anticipation of novelty recruits reward system and hippocampus while promoting recollection. *NeuroImage*, *38*(1), 194–202. <https://doi.org/10.1016/j.neuroimage.2007.06.038>

Wolf, D. H., Turetsky, B. I., Loughead, J., Elliott, M. A., Pratiwadi, R., Gur, R. E., & Gur, R. C. (2008). Auditory Oddball fMRI in Schizophrenia: Association of Negative Symptoms with Regional Hypoactivation to Novel Distractors. *Brain Imaging and Behavior*, *2*(2), 132–145. <https://doi.org/10.1007/s11682-008-9022-7>

Yamaguchi, S. (2004). Rapid Prefrontal-Hippocampal Habituation to Novel Events. *Journal of Neuroscience*, *24*(23), 5356–5363. <https://doi.org/10.1523/JNEUROSCI.4587-03.2004>

Zäske, R., Awwad Shiekh Hasan, B., & Belin, P. (2017). It doesn’t matter what you say: FMRI correlates of voice learning and recognition independent of speech content. *Cortex*, *94*, 100–112. <https://doi.org/10.1016/j.cortex.2017.06.005>
